# A non-canonical pathway regulates ER stress signaling and blocks ER stress-induced apoptosis and heart failure

**DOI:** 10.1038/s41467-017-00171-w

**Published:** 2017-07-25

**Authors:** Yufeng Yao, Qiulun Lu, Zhenkun Hu, Yubin Yu, Qiuyun Chen, Qing K. Wang

**Affiliations:** 1 Key Laboratory of Molecular Biophysics of the Ministry of Education, College of Life Science and Technology and Center for Human Genome Research, Huazhong University of Science and Technology, Wuhan, Hubei Province 430074 China; 2 Department of Molecular Cardiology, Center for Cardiovascular Genetics, Cleveland Clinic, Cleveland, OH 44195 USA; 3 Department of Molecular Medicine, CCLCM, Case Western Reserve University, Cleveland, OH 44195 USA; 4 Department of Genetics and Genome Sciences, Case Western Reserve University, Cleveland, OH 44195 USA

## Abstract

Endoplasmic reticulum stress is an evolutionarily conserved cell stress response associated with numerous diseases, including cardiac hypertrophy and heart failure. The major endoplasmic reticulum stress signaling pathway causing cardiac hypertrophy involves endoplasmic reticulum stress sensor PERK (protein kinase-like kinase) and eIF2α-ATF4-CHOP signaling. Here, we describe a non-canonical, AGGF1-mediated regulatory system for endoplasmic reticulum stress signaling associated with increased p-eIF2α and ATF4 and decreased sXBP1 and CHOP. Specifically, we see a reduced AGGF1 level consistently associated with induction of endoplasmic reticulum stress signaling in mouse models and human patients with heart failure. Mechanistically, AGGF1 regulates endoplasmic reticulum stress signaling by inhibiting ERK1/2 activation, which reduces the level of transcriptional repressor ZEB1, leading to induced expression of miR-183-5p. miR-183-5p post-transcriptionally downregulates CHOP and inhibits endoplasmic reticulum stress-induced apoptosis. AGGF1 protein therapy and miR-183-5p regulate endoplasmic reticulum stress signaling and block endoplasmic reticulum stress-induced apoptosis, cardiac hypertrophy, and heart failure, providing an attractive paradigm for treatment of cardiac hypertrophy and heart failure.

## Abstract (web summary)

Endoplasmic reticulum (ER) stress promotes cardiac dysfunction. Here the authors uncover a pathway whereby AGGF1 blocks ER stress by inhibiting ERK1/2 activation and the transcriptional repressor ZEB1, leading to induction of miR-183-5p and down-regulation of CHOP, and show that AGGF1 can effectively treat cardiac hypertrophy and heart failure.

## Introduction

Endoplasmic reticulum (ER) stress is an evolutionarily conserved cell stress response and has been associated with numerous diseases, including cardiovascular diseases, Alzheimer’s disease, Parkinson’s disease, Huntington’s disease, diabetes, renal failure, fatty liver disease, irritable bowel syndrome, and many others^1–9^. ER stress plays a critical role in the development of pathological cardiac hypertrophy and heart failure, a major public health issue with a prevalence of 23 million worldwide, 2.4 million hospitalization, and > 300,000 deaths each year in the United States alone^10–14^.

ER is the primary site of protein synthesis, folding, and secretion^15, 16^. Many factors such as ischemia, hypoxia, heat shock, genetic mutation, oxidative stress, and increased protein synthesis impair the functions of ER^17^. Perturbations in ER function are referred to as “ER stress”, which leads to the accumulation of unfolded and misfolded proteins in the ER, and triggers the unfolded protein response^18–20^. ER accumulation of unfolded proteins is detected by at least three ER transmembrane sensors, including PERK (protein kinase-like kinase), ATF6, and IRE1, which activate signaling pathways of PERK-eIF2α-ATF4-CHOP, pro-ATF6 to cleaved ATF6 (cATF6) and CHOP, and IRE1-spliced XBP1 (sXBP1), respectively^8, 19, 21–23^. This leads to upregulation of the genes for ER chaperones and ER-associated degradation components^20^. When ER stress is prolonged or severe, it induces apoptosis to eliminate unhealthy cells, contributing to the process of cardiac hypertrophy^8, 24, 25^. Prolonged cardiac hypertrophy causes dilated cardiomyopathy, heart failure, arrhythmias, and sudden death^26, 27^.

ER stress induces the apoptotic pathway by stimulating the transcriptional activation of pro-apoptotic transcriptional factor C/EBP homologous protein (CHOP), also known as growth arrest and DNA damage-inducible gene 153 (GADD153)^28–30^. CHOP is induced during ER stress^31, 32^. Transcriptional activation of CHOP is induced by activating transcription factor 4 (ATF4), whereas during ER stress only specific mRNAs, such as the *ATF4* and *CHOP* mRNA, are translated because of increased eIF2α phosphorylation^8, 33^. ATF4 binds to the *CHOP* promoter directly and activates its transcription^29^. On the other hand, the levels of ATF4 and phosphorylated eIF2α (p-eIF2α) can be reduced by CHOP through a negative feedback regulatory mechanism via activating the transcription of the *GADD34* gene encoding a regulatory subunit of the phosphatase for eIF2α^24, 28, 34^. The level of *CHOP* was significantly induced in cardiac hypertrophy and heart failure^24, 29^. *Chop* ^*−/−*^ knockout (KO) mice showed attenuated cardiac hypertrophy and dysfunction induced by pressure overload and reduced apoptosis in response to ER stress^24^. CHOP overexpression leads to cell cycle arrest or induces apoptosis by regulating the abundance of multiple pro-apoptotic factors, including death receptor 5 (DR5), Tribbles homolog 3 (TRB3), and p53-upregulated modulator of apoptosis (Puma)^35–37^. CHOP also contributes to apoptosis through regulating ERO1α, an ER oxidase that promotes hyperoxidization of the ER^28^. Through dimerization with cAMP-responsive element binding protein, CHOP can suppress the transcription of the survival protein Bcl2^35^. Thus, CHOP is involved in ER stress-induced cardiac apoptosis, hypertrophy, and heart failure.

The *AGGF1* gene encodes an AngioGenic factor with a G-patch domain and a Forkhead-associated domain (FHA) 1 and confers risk of a congenital vascular disorder Klippel-Trenaunay syndrome^38, 39^. Recombinant AGGF1 stimulates angiogenesis as potently as vascular endothelial growth factor A (VEGFA)^38, 40^. AGGF1 was also found to inhibit ischemia-reperfusion-induced cardiac apoptosis^41^. During zebrafish embryogenesis, *Aggf1* is required for differentiation of multipotent hemangioblasts^42^ and determination of veins^43^. Our recent studies showed that Aggf1 is essential for early embryogenesis and vascular development, physiological and pathological angiogenesis, and inhibition of vascular permeability^44^. AGGF1 can induce autophagy by activating JNK, regulate angiogenesis and vascular development by activating PI3K, AKT, GSK3β, and S6K and by inhibiting ERK, and maintain vascular integrity by inhibiting vascular endothelial ca﻿dherin (VE-cadherin) phosphorylation^44, 45^. Most interestingly, AGGF1 protein therapy can robustly restore cardiac function in a myocardial infarction model and an ischemia-reperfusion model^44, 45^.

In this study, we investigated the physiological role of *Aggf1* in the heart using *Aggf1* ^*+/−*^ mice and unraveled a novel role of *Aggf1* in regulating ER stress signaling, cardiac hypertrophy, and heart failure. We discovered a noncanonical signaling pathway, AGGF1-ERK-ZEB1-miR-183-5p-CHOP, that blocks ER stress-induced apoptosis and cardiac hypertrophy. Our data suggest that AGGF1 regulates ER stress signaling pathways, and blocks ER stress-induced apoptosis, cardiac hypertrophy, and heat failure.

## Results

### *Aggf1* haploinsufficiency induces cardiac hypertrophy

**Fig. 1:**
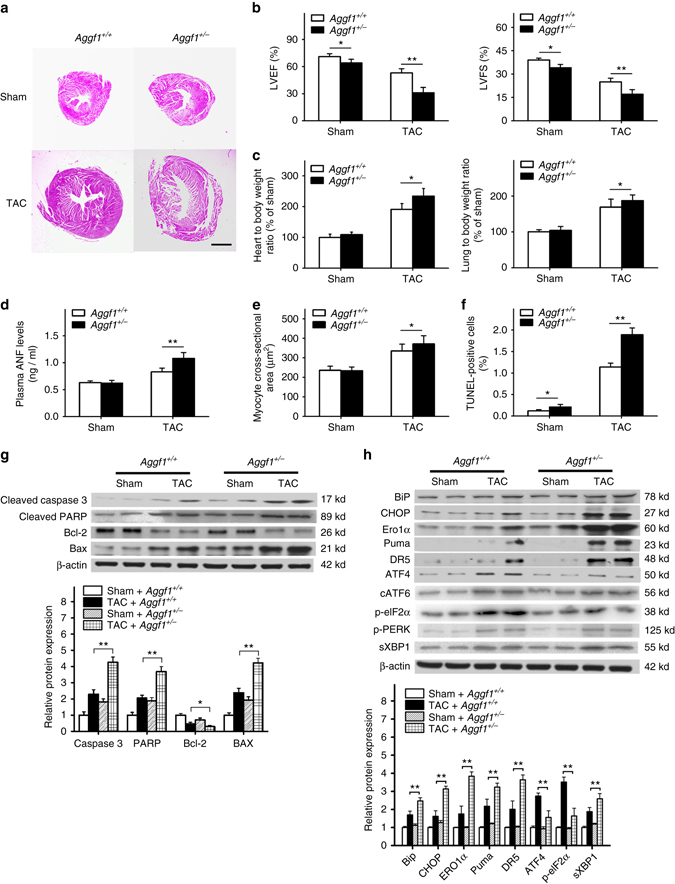
In vivo role of *Aggf1* in ER stress, cardiac apoptosis, and hypertrophy. TAC models for cardiac hypertrophy and heart failure were created for both wild-type and *Aggf1* ^*+/−*^ male mice and characterized 6 weeks after surgeries. Sham, the same surgical procedure as TAC but without aortic constriction. **a** Representative H&E staining images of heart sections (*n* = 12/group). *Scale bar*, 1 mm. **b** LVEF and LVFS (*n* = 12/group, *P < 0.05, **P < 0.01). **c** Ratios of heart weight to body weight (*n* = 12/group, **P < 0.01) and lung weight to body weight (*n* = 12/group, *P < 0.05). **d** ELISA assays for the plasma ANF level (*n* = 12/group, **P < 0.01). **e** Cross-sectional diameter (µm) of cardiomyocytes (*n* = 12/group, *P < 0.05). **f** Quantitative analysis of TUNEL-positive myocardial cells (*n* = 6/group, **P < 0.01). **g** Western blot analysis for markers for apoptosis (*n* = 6/group, *P < 0.05, **P < 0.01). **h** Western blot analysis for ER stress signaling markers using cardiac protein extracts, including cATF6 and sXBP1 (*n* = 6/group, *P < 0.05, **P < 0.01). Data are presented as the mean ± s.d. from at least three independent experiments. Statistical analysis was carried out by a Student’s two-tailed *t*-test

We investigated whether AGGF1 had a role in cardiac function by characterizing *Aggf1* ^*+/−*^ mice, which contains a gene-trapping allele with a splicing acceptor and a stop codon in intron 11 of *Aggf1* ^44^. Western blot analysis showed a 50% decrease of the level of the AGGF1 protein in *Aggf1* ^*+/−*^ mice (referred to as haploinsufficiency)^44^. Because *Aggf1* ^*−/−*^ mice are embryonically lethal, we studied *Aggf1* ^*+/−*^ mice. There were no significant differences in systolic blood pressure (SBP) between wild-type (WT) mice and *Aggf1* ^*+/−*^ mice under the physiological condition (108.3 ± 12.5 vs. 114.4 ± 13.7 mm Hg; *P* = 0.41 Student’s *t*-test). No significant difference was found for the heart rate between the two groups of mice, either (WT: 628 ± 35 vs. *Aggf1* ^*+/−*^: 589 ± 48 bpm; *P* = 0.09 Student’s *t*-test). We induced cardiac hypertrophy and heart failure in *Aggf1* ^*+/−*^ mice using transverse aortic constriction (TAC), a well-established model for cardiac hypertrophy and heart failure in small animals. Six weeks after TAC, *Aggf1* ^*+/−*^ mice showed more severe cardiac hypertrophy than WT mice by hematoxylin and eosine (H&E) staining (Fig. 1a). Echocardiography showed that TAC induced significantly more reduction of left ventricular ejection fraction (LVEF) and LV fraction shortening (LVFS) in *Aggf1* ^*+/−*^ mice than in WT mice (Fig. 1b). Consistent with this finding, after TAC, the ratio of heart weight to body weight (HW/BW) and the ratio of lung weight to BW (LW/BW) were increased significantly more in *Aggf1* ^*+/−*^ mice than in WT mice (Fig. 1c). The cross-sectional diameter of cardiomyocytes and the plasma level of atrial natriuretic factor (ANF), a sensitive biomarker for cardiac hypertrophy and heart failure, were increased more in *Aggf1* ^*+/−*^ mice than in WT mice after TAC (Fig. 1d, e). *Aggf1* ^*+/−*^ mice also showed an increased level of hypertrophic markers *Nppa* encoding ANF or *Nppb* encoding BNP after TAC (Supplementary Fig. 1). These data suggest that *Aggf1* haploinsufficiency significantly augments cardiac hypertrophy and heart failure in a TAC model.

The cardiac function (LVEF and LVFS) was significantly impaired in heterozygous *Aggf1* ^*+/−*^ mice and control WT mice in the sham group, although the effect was small (Fig. 1b), which may be due to the reduced angiogenesis in *Aggf1* ^*+/−*^ mice reported previously^44, 45^. No significant differences were found for other parameters of the cardiac structure and function between *Aggf1* ^*+/−*^ mice and control WT mice in the sham group (Fig. 1c–e).

### *Aggf1* haploinsufficiency induces ER stress-induced apoptosis

TdT-mediated dUTP nick end labeling (TUNEL) staining of heart sections showed that TAC induced significantly more increases in the number of apoptotic myocardial cells in *Aggf1* ^*+/−*^ mice than in WT mice after TAC (Fig. 1f). *Aggf1* ^*+/−*^ mice showed an enhanced level of myocardial fibrosis induced by TAC (Supplementary Fig. 2). Western blot analysis with heart extracts showed that TAC induced higher levels of cleaved caspase3, cleaved PARP, and Bax, but a lower level of Bcl-2 in *Aggf1* ^*+/−*^ mice than in WT mice after TAC (Fig. 1g). These data suggest that increased apoptosis by *Aggf1* haploinsufficiency may be responsible for augmented cardiac hypertrophy and heart failure.

ER stress induces apoptosis through the CHOP pathway^24, 29^. To further identify the mechanism by which *Aggf1* haploinsufficiency augments cardiac hypertrophy and heart failure, we analyzed CHOP and other ER stress signaling markers BiP, ERO1α, Puma, DR5, ATF4, cATF6, p-eIF2α, phosphorylated PERK (p-PERK), and sXBP1 using western blot analysis. *Aggf1* haploinsufficiency induced significantly more increases in the abundance of ER stress signaling markers BiP, CHOP, ERO1α, Puma, DR5, and sXBP1 in the TAC group (Fig. 1h). No significant differences were found for the abundance of cATF6 and p-PERK (Fig. 1h). Surprisingly, for ER stress signaling markers ATF4 and p-eIF2α, an unexpected finding was made. TAC induced significantly higher levels of ATF4 and p-eIF2α in WT mice, which was abolished in *Aggf1* ^*+/−*^ mice (Fig. 1h). These data suggest that *Aggf1* haploinsufficiency regulates ER stress signaling, which may be responsible for increased apoptosis and augmented cardiac hypertrophy and heart failure after TAC.

### AGGF1 downregulation and ER stress signaling in patients

**Fig. 2:**
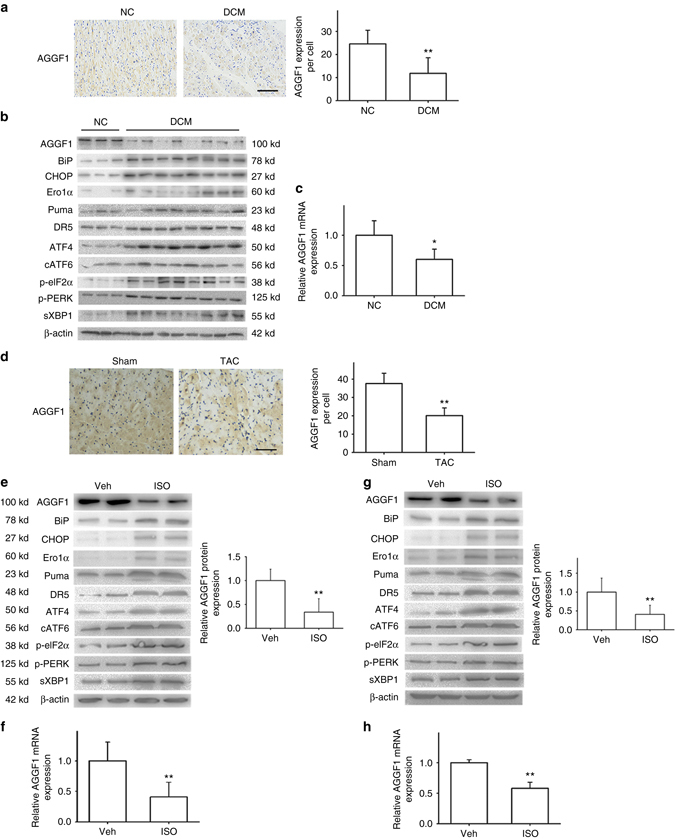
Association of decreased AGGF1 and increased ER stress signaling. **a** Representative immunohistochemical images for AGGF1 expression (*left*) in heart sections from eight dilated cardiomyopathy (DCM) patients and three controls (NC) and quantification (*right*; **P < 0.01). *Scale bar*, 100 μm. **b** Western blot analysis for AGGF1 and ER stress signaling markers using protein extracts from eight DCM patients and three controls (NC group: *n* = 3; DCM group: *n* = 8, **P < 0.01). **c** Real-time RT-PCR analysis for the level of *AGGF1* mRNA in patients’ hearts (*P < 0.05). **d** Representative immunohistochemical images for AGGF1 expression (*left*) in heart sections from TAC mice vs. control Sham mice (12 weeks of age, 20–25 g) and quantification (*right*; *n* = 5/group, **P < 0.01). *Scale bar*, 50 μm. **e** Western blot analysis for AGGF1 and ER stress signaling markers using protein extracts from mice with ISO treatment vs. mice with control vehicle (Veh) treatment (*n* = 6/group, **P < 0.01). **f** Real-time RT-PCR analysis for the level of *AGGF1* mRNA using cDNA extracts from mice with ISO treatment vs. mice with control vehicle (Veh) treatment (*right*; *n* = 5/group, **P < 0.01). **g** Western blot analysis for AGGF1 and ER stress signaling markers using protein extracts from H9C2 cells treated with or without ISO (*n* = 6/group, **P < 0.01). **h** Real-time RT-PCR analysis for the level of *AGGF1* mRNA using RNA extracts from H9C2 cells treated with or without ISO (*n* = 3/group, **P < 0.01). Data are shown as the mean ± s.d. from at least three independent experiments. Statistical analysis was carried out by a Student’s two-tailed *t*-test

Immunostaining analysis of heart sections showed that the level of AGGF1 was significantly decreased in patients with heart failure compared with normal controls (Fig. 2a). Both western blot and real-time reverse transcriptase polymerase chain reaction (RT-PCR) analyses showed that the level of AGGF1 was decreased in patients compared with normal controls (Fig. 2b, c). We also found that the level of CHOP was significantly higher in the hearts of patients with heart failure (Fig. 2b). Similarly, other ER stress signaling markers were also higher in patient samples than in control hearts (Fig. 2b). These data revealed an interesting association between downregulation of AGGF1 and increased ER stress signaling in human patients with heart failure.

### AGGF1 and ER stress signaling in cellular and animal models

The association between downregulation of AGGF1 and increased ER stress signaling was also identified in a TAC model for cardiac hypertrophy and heart failure in 12-week-old C57BL/6 male mice (Figs. 1h and 2d).

In mice pumped with isoproterenol (ISO) or control vehicle for 4 weeks, the AGGF1 protein level was decreased, whereas the levels of CHOP and other ER stress signaling markers were increased in ISO-treated heart tissues (Fig. 2e). Moreover, the level of *Aggf1* mRNA in cardiac tissue samples was decreased after ISO treatment (Fig. 2f).

We also developed a cellular model relevant to cardiac hypertrophy by incubating H9C2 cells (a rat cardiomyoblast cell line) with ISO for 48 h. ISO decreased the levels of *Aggf1* mRNA and protein, which was accompanied by an increased level of CHOP and other ER stress signaling markers (Fig. 2g, h).

### AGGF1 protein therapy attenuates cardiac hypertrophy

**Fig. 3:**
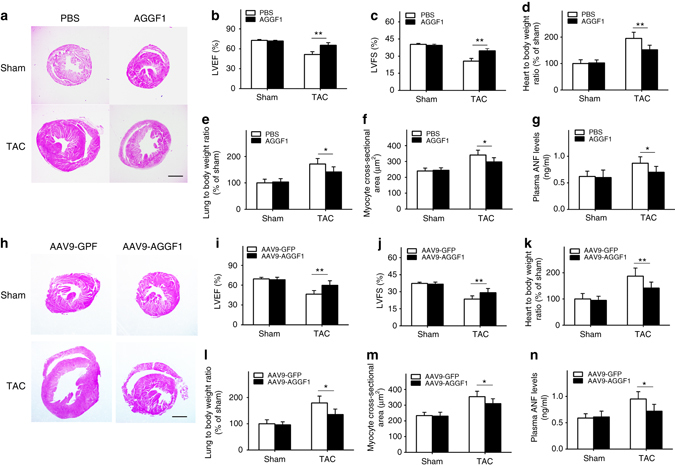
AGGF1 therapy of TAC mice with cardiac hypertrophy. **a**–**g** AGGF1 protein therapy; **h**–**n** AGGF1 gene therapy using AAV9 viruses (*n* = 12 per group, *P < 0.05, **P < 0.01). **a**, **h** H&E staining images of heart tissues. *Scale bar*, 1 mm. (**b**, **i**) Echocardiographic data on LVEF. **c**, **j** Echocardiographic data on LVFS. **d**, **k** The ratio of heart weight to body weight. **e**, **l** The ratio of lung weight to body weight. **f**, **m** The cross-sectional diameter (µm) of cardiomyocytes. **g**, **n** Plasma ANF levels. Data are presented as the mean ± s.d. from at least three independent experiments. Statistical analysis was carried out by a Student’s two-tailed *t*-test

As AGGF1 abundance is reduced in patients and animal models with heart failure, we explored the potential of therapeutic treatment of cardiac hypertrophy and heart failure by targeted protein therapy via intravenous injection of recombinant AGGF1 (0.25 mg/kg BW). We utilized 6-week-old TAC mice as a small animal model for AGGF1 protein therapy. As shown in Fig. 3a, H&E staining showed that TAC induced cardiac hypertrophy, but AGGF1 protein therapy resulted in less enlargement of the hearts compared with phosphate-buffered saline (PBS) treatment. Echocardiography showed that AGGF1 treatment improved cardiac functions in TAC mice by restoring LVEF and LVFS to the same levels as in control sham mice (Fig. 3b, c). Similarly, AGGF1 therapy significantly reduced the ratios of HW/BW and LW/BW, and the cross-sectional diameter of cardiomyocytes in TAC mice compared (Fig. 3d–f). AGGF1 also inhibited TAC-induced myocardial fibrosis (Supplementary Fig. 3). The plasma level of ANF was also significantly lower in the AGGF1 treatment group than in the PBS treatment group (Fig. 3g). AGGF1 protein treatment significantly decreased the levels of *Nppa* and *Nppb* mRNA after TAC (Supplementary Fig. 4). AGGF1 protein therapy did not affect the heart rate (PBS: 611 ± 36 vs. AGGF1: 573 ± 42 bpm; *P* = 0.07, Student’s *t*-test) or SBP (PBS: 117.9 ± 16.6 vs. AGGF1: 109.2 ± 19.1 mm Hg; *P* = 0.32, Student’s *t*-test). These data suggest that AGGF1 protein therapy can successfully inhibit cardiac hypertrophy and heart failure and improve cardiac function. Interestingly, the AGGF1 protein therapy increased the ventricular angiogenesis. Immunostaining with an anti-CD31 antibody (an endothelial cell marker) with sections from left ventricles showed more CD31-positive vessels in TAC mice treated with the AGGF1 protein than those treated with control PBS or sham mice (Supplementary Fig. 5).

In order to confirm our finding that AGGF1 protein therapy can successfully inhibit cardiac hypertrophy and heart failure and improve cardiac function, we injected adeno-associated viruses AAV9-AGGF1 into the myocardium of mice to drive the ectopic expression of AGGF1 in myocardial cells (AAV9-GFP viruses as control). TAC was then created and the mice were analyzed 6 weeks later. *AGGF1* overexpression inhibited cardiac hypertrophy, improved cardiac function, and reduced the ratios of HW/BW and LW/BW, and the cross-sectional diameter of cardiomyocytes (Fig. 3h–n). AAV9-AGGF1 also inhibited TAC-induced myocardial fibrosis (Supplementary Fig. 6). Together, these data demonstrate that *AGGF1* overexpression by gene delivery via AAV9 viruses can successfully inhibit cardiac hypertrophy and improve cardiac function.

### AGGF1 protein therapy regulates ER stress signaling

**Fig. 4:**
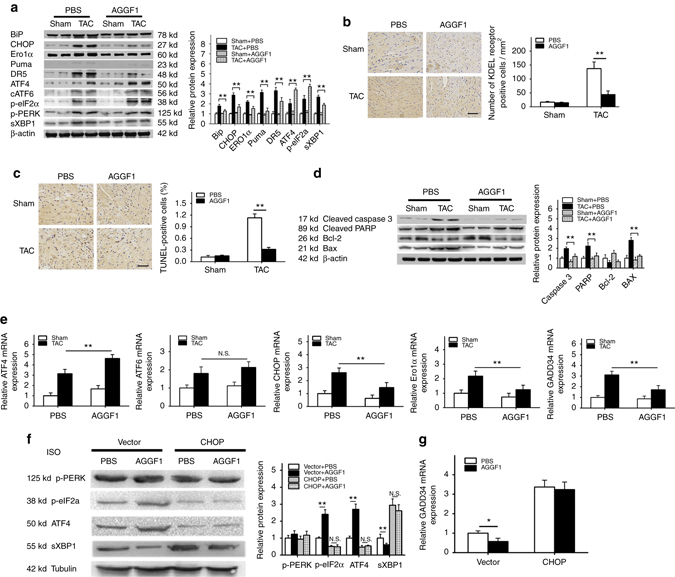
AGGF1 protein therapy regulates ER stress signaling and apoptosis. TAC or sham mice were treated with AGGF1 or PBS (*left*) and characterized (*n* = 6/group, **P < 0.01). **a** AGGF1 regulates TAC-induced ER stress signaling in mice. Protein extracts from heart samples were used for western blot analysis for ER stress signaling markers (*n* = 6/group, **P < 0.01). **b** Representative images for immunostaining analysis of heart sections for KDEL receptor-positive cells. *Scale bar*, 50 μm. **c** Representative images of TUNEL staining for apoptosis from heart sections. *Scale bar*, 50 μm. **d** Western blot analysis for apoptosis markers in heart tissues (*n* = 6/group, **P < 0.01). **e** Real-time RT-PCR analyses for *ATF4*, *ATF6*, *CHOP*, *Ero1α*, and *GADD34* in heart tissues from TAC or sham mice treated with AGGF1 or PBS (*n* = 5/group, **P < 0.01). **f** Western blot analysis showing that AGGF1 protein treatment increased the levels of ATF4 and p-eIF2α, and decreased the level of sXBP1 in H9C2 cells treated with ISO for 48 h. The effects of AGGF1 were abolished by overexpression of *CHOP* by transient transfection of an expression plasmid as compared with the empty vector. No effect was observed for p-PERK (*n* = 3/group, **P < 0.01, *N.S.*, Non-significant). **g** Real-time RT-PCR analysis for *GADD34* in H9C2 cells transfected with an expression plasmid for *CHOP* or empty vector control, and then treated with ISO in combination with AGGF1 or PBS for 48 h (*n* = 3/group, *P < 0.05). Data are shown as the mean ± s.d. from at least three independent experiments. For **a**–**e**, statistical analysis was carried out by a Student’s two-tailed *t*-test; for **f**, **g**, statistical analysis was carried out by one-way analysis of variance

As shown in Fig. 4a, ER stress was induced by TAC after 6 weeks as shown by increased levels of BiP, CHOP, ERO1a, Puma, DR5, ATF4, cATF6, sXBP1, p-eIF2α, and p-PERK. Intravenous injection of AGGF1 protein suppressed ER stress signaling as shown by decreased levels of CHOP, ERO1a, Puma, DR5, and sXBP1 (Fig. 4a). We also analyzed ER stress signaling by immunostaining analysis of the left ventricular sections for the KDEL receptor (Fig. 4b). The KDEL receptor is a retrieval receptor for ER chaperones in the early secretary pathway, and transgenic overexpression of a mutant KDEL receptor disrupts the recycling of misfolded proteins between ER and Golgi, increases the CHOP level and apoptosis, and results in dilated cardiomyopathy^46^. TAC induced ER stress signaling, which was greatly suppressed by AGGF1 treatment (Fig. 4b). The ER stress activator tunicamycin (TM) increased the abundance of CHOP, ERO1α, and DR5, which was suppressed by AGGF1 treatment (Supplementary Fig. 7).

Severe or prolonged ER stress triggers apoptosis^28^. TUNEL staining of heart sections showed that TAC induced apoptosis, whereas AGGF1 treatment significantly inhibited apoptosis (Fig. 4c). We also used western blot analysis to examine characteristic markers for apoptosis, and found that TAC increased the abundance of cleaved PARP, cleaved caspase-3, and Bax, all of which were abolished by AGGF1 treatment (Fig. 4d).

Some of changes associated with markers for ER stress signaling and ER stress-induced apoptosis were also confirmed using real-time RT-PCR analysis at the mRNA level. As shown in Fig. 4e and Supplementary Fig. 8, AGGF1 treatment significantly decreased the increased levels of *CHOP*, *ERO1α*, and spliced *XPB1* induced by TAC. The level of *ATF6* mRNA was increased by TAC; however, the effect was not affected by the AGGF1 protein treatment (Fig. 4e). In the canonical signaling pathway, ER stress was associated with increased abundance of ATF4, a transcriptional factor that directly activates the transcription of CHOP and follow-up ER stress^29^. As AGGF1 regulates ER stress signaling, we expected it to decrease ATF4 abundance. However, we made an unexpected finding in which AGGF1 induced ATF4 expression both at the protein level (Fig. 4a) and at the mRNA level (Fig. 4e). This may be caused by a negative feedback regulatory mechanism through which AGGF1 reduces the level of CHOP, which then decreases transcriptional activation of *GADD34*, resulting in an increased level of p-eIF2α and ATF4^24, 28, 34^. Consistent with the hypothesis, AGGF1 decreased the level of *GADD34* in the TAC group (Fig. 4e).

In ISO-treated H9C2 cells, AGGF1 treatment increased the levels of p-eIF2α and ATF4; however, the effects were abolished by overexpression of CHOP (Fig. 4f). AGGF1 treatment decreased the level of *GADD34* (Fig. 4g); however, the effect was abolished by overexpression of CHOP (Fig. 4f, g). The level of sXBP1 was reduced by AGGF1 treatment; however, the effect was abolished by overexpression of CHOP (Fig. 4f). No effects were observed for p-PERK (Fig. 4f). These data suggest that AGGF1 reduces the level of CHOP, and the reduced CHOP level decreased the level of GADD34, which increases the levels of p-eIF2α and ATF4.

### Novel mechanism by which AGGF1 regulates ER stress signaling

**Fig. 5:**
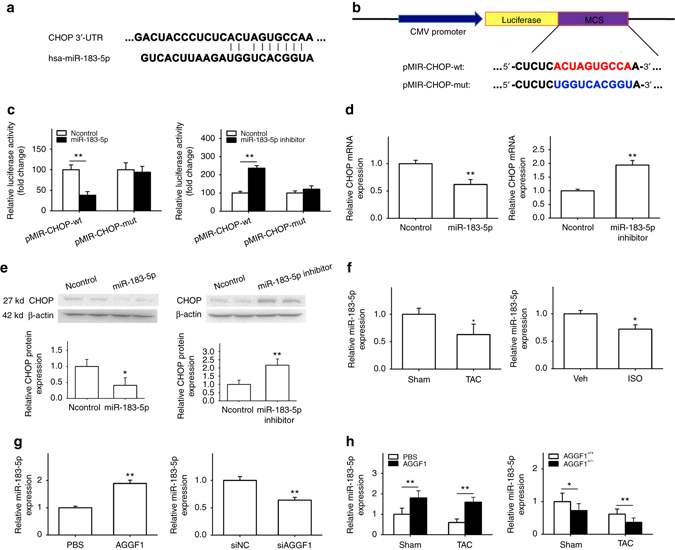
AGGF1 downregulates expression of *CHOP* via *miR-183-5p*. **a** Bioinformatic analysis of the *CHOP* mRNA sequence identified a binding site for *miR-183-5p* at the 3′-UTR. **b** *Schematic diagram* showing the wild type (wt) pMIR-CHOP-wt or mutant pMIR-CHOP-mut reporter with the miR-183-5p-binding site mutated. **c** Luciferase activity of pMIR-CHOP-wt or mutant pMIR-CHOP-mut reporters in the presence of miR-183-5p mimics, a negative control miRNA mimics (Ncontrol), or a miR-183-5p specific inhibitor (*n* = 3/group, **P < 0.01). **d** Quantitative real-time RT-PCR analysis for the level of the *CHOP* mRNA in H9C2 cells with transfection of miR-183-5p mimics or a miR-183-5p-specific inhibitor compared with Ncontrol (*n* = 3/group, **P < 0.01). **e** Western blot analysis for CHOP abundance in H9C2 cells with transfection of miR-183-5p mimics or a miR-183-5p*-*specific inhibitor compared with Ncontrol. **f** Real-time RT-PCR analysis showing decreased miR-183-5p expression in TAC mice (*n* = 5/group, *P < 0.05) or in an ISO-induced H9C2 cell model for cardiac hypertrophy (*n* = 3/group, *P < 0.05). **g** Real-time RT-PCR analysis for miR-183-5p expression in H9C2 cells 48 h after AGGF1 treatment (*left*, *n* = 3/group, **P < 0.01). Real-time RT-PCR analysis for miR-183-5p expression in H9C2 cells transfected with *AGGF1-*specific siRNA (siAGGF1) or siNC (*right*, *n* = 3/group, **P < 0.01). **h** Real-time RT-PCR analysis for miR-183-5p expression in the hearts of TAC or Sham mice with or without AGGF1 treatment (*left*, *n* = 6/group, **P < 0.01). Real-time RT-PCR analysis for miR-183-5p expression in the hearts of WT (*Aggf1* ^*+/+*^) or *Aggf1* ^*+/−*^ mice after TAC or sham surgeries (*right*, *n* = 6/group, *P < 0.05, **P < 0.01). Data are shown as the mean ± s.d. from at least three independent experiments. Statistical analysis was carried out by a Student’s two-tailed *t*-test

To identify the molecular mechanism by which AGGF1 regulates ER stress signaling, we explored a post-transcriptional regulatory mechanism that AGGF1 reduces the level of the key ER stress regulator, CHOP, by microRNA targeting the 3′-untranslated region (UTR) of *CHOP*. We used TargetScan (http://www.targetscan.org/) to analyze the 3′-UTR of *CHOP* for a potential microRNA-binding site. The analysis revealed a potential binding site for miR-183-5p at the 3′-UTR of *CHOP* (Fig. 5a). Luciferase reporters were created for the binding site (pMIR-CHOP-wt and pMIR-CHOP-mut with the site mutated; Fig. 5b). Transfection of miR-183-5p mimics significantly reduced luciferase activities from the WT reporter compared to control miRNA mimics, an effect that was abolished in the mutant reporter in which the miR-183-5p-binding site was mutated (Fig. 5c and Supplementary Fig. 9). Transfection of the miR-183-5p inhibitor increased luciferase activities from the WT reporter, whereas this effect was abolished in the case of the mutant reporter (Fig. 5c). These data suggest that miR-183-5p negatively regulates the expression of *CHOP* by directly binding to the 3′-UTR of *CHOP*.

In order to further confirm the above results, we assessed the levels of *Chop* mRNA using real-time RT-PCR analysis in H9C2 cells transfected with miR-183-5p mimics or non-target negative control (Ncontrol) miRNA mimics (Fig. 5d). Compared to control mimics, miR-183-5p mimics significantly reduced the mRNA level of *Chop* (Fig. 5d). Moreover, the miR-183-5p inhibitor significantly increased the level of *Chop* mRNA. Western blot analysis showed that the level of the CHOP protein was significantly decreased by miR-95-3p mimics and increased by miR-183-5p inhibitor (Fig. 5e). Together, all these data again indicate that miR-183-5p post-transcriptionally downregulates *Chop*.

The level of miR-183-5p was significantly decreased in TAC mice (Fig. 5f). Similarly, in H9C2 cells treated with ISO (a cell model for hypertrophy), the level of miR-183-5p was also significantly decreased (Fig. 5f). We examined the level of CHOP in TAC mice with or without Ago-miR-183-5p mimics. The Ago-miR-183-5p mimics significantly reduced the level of CHOP (Supplementary Fig. 10). We also examined the level of CHOP in TAC mice with or without Antago-miR-183-5p inhibitor. The Antago-miR-183-5p inhibitor increased CHOP expression in TAC mice (Supplementary Fig. 10).

We then analyzed whether AGGF1 regulates the level of miR-183-5p. Treatment of H9C2 myoblasts with AGGF1 significantly increased the level of miR183-5p (Fig. 5g), whereas *AGGF1*-specific short interfering RNA (siRNA) significantly reduced the level of miR183-5p compared with control siRNA (siNC; Fig. 5g). The efficiency of AGGF1 siRNA was confirmed using real-time RT-PCR analysis (Supplementary Fig. 11). Moreover, real-time RT-PCR analysis showed that the level of miR-183-5p was decreased in the hearts from TAC mice compared to control mice with sham operation. Either in WT mice or in TAC mice, AGGF1 treatment increased the level of miR-183-5p (Fig. 5h). More interestingly, AGGF1 treatment significantly rescued the loss of miR-183-5p expression caused by TAC (Fig. 5h). Furthermore, the level of miR-183-5p was significantly decreased in *Aggf1* ^*+/−*^ mice with or without TAC (Fig. 5h).

**Fig. 6:**
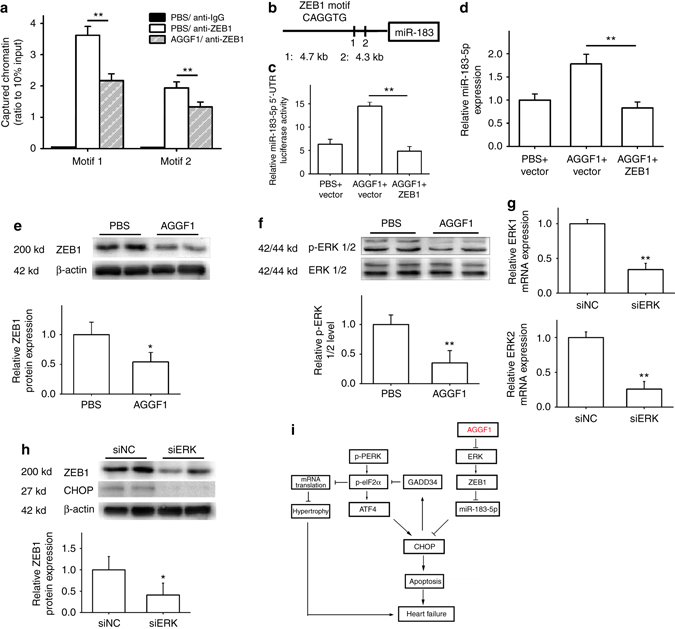
Mechanism for AGGF1 regulation of *miR-183-5p* expression. **a** ChIP–qPCR analysis for the interaction between ZEB1 and miR-183-5p promoter DNA (*n* = 4/group, **P < 0.01). **b** *Schematic diagram* showing that the miR-183-5p promoter-luciferase reporter with two ZEB1-binding motifs. **c** Luciferase activity (*n* = 6/group, **P < 0.01). **d** Real-time RT-PCR analysis for miR-183-5p expression in H9C2 cells after AGGF1 treatment with or without *ZEB1* overexpression (*n* = 3/group, **P < 0.01). **e** Western blot analysis for the ZEB1 level in H9C2 cells after AGGF1 treatment (*n* = 5/group, *P < 0.05). **f** Western blot analysis for activation of ERK1/2 in H9C2 cells after AGGF1 treatment (*n* = 5/group, **P < 0.01). **g** Real-time RT-PCR analysis for *ERK1* and *ERK2* expression by ERK siRNA (*n* = 3/group, **P < 0.01). **h** Western blot analysis for the ZEB1 level by ERK siRNA (*n* = 5/group, *P < 0.05). **i** *Schematic model* for a non-canonical ER stress signaling pathway mediated by AGGF1. Data are shown as the mean ± s.d. from at least three independent experiments. Statistical analysis was carried out by a Student’s two-tailed *t*-test

To identify the molecular mechanism by which AGGF1 regulates the level of miR-183-5p, we analyzed the promoter/regulatory region of miR-183-5p and found that it contained two binding sites for a transcriptional repressor, Zinc Finger E-Box Binding Homeobox 1 (ZEB1)^47, 48^. Our chromatin immunoprecipitation (ChIP) analysis showed that ZEB1 was able to bind to both sites (Fig. 6a). We constructed a miR-183-5p promoter/luciferase reporter gene (Fig. 6b). Luciferase assays showed that AGGF1 treatment increased the luciferase activity from the reporter gene, and overexpression of *ZEB1* abolished the stimulatory effect of AGGF1 on the reporter in H9C2 cells (Fig. 6c). Quantitative RT-PCR analysis also showed that AGGF1 treatment increased the level of miR-183-5p, but overexpression of *ZEB1* abolished the stimulatory effect of AGGF1 on the level of miR-183-5p (Fig. 6d). We used siRNA to knock *ZEB1* expression down and determined whether it can rescue the effect of AGGF1 knockdown. On the basis of our model, *AGGF1* knockdown reduces the level of miR-183-5p due to increased *ZEB1*; therefore, *ZEB1* knockdown is expected to rescue the effect of *AGGF1* knockdown. Our luciferase assays showed that siAGGF1 decreased the luciferase activity from the miR-183-5p promoter luciferase reporter (pGL3-miR-183-5p), and siZEB1 abolished the effect of *AGGF1* knockdown on the reporter in H9C2 cells (Supplementary Fig. 12). Quantitative RT-PCR analysis also showed that siAGGF1 decreased the level of miR-183-5p, but siZEB1 abolished the effect of *AGGF1* on the level of miR-183-5p. These data suggest that *ZEB1* acts downstream of *AGGF1* in regulation of the level of miR-183-5p (Supplementary Fig. 12).

We then determined whether AGGF1 regulates the level of *ZEB1*. Western blot analysis showed that AGGF1 treatment significantly reduced the level of ZEB1 (Fig. 6e). Consistent with this finding, ChIP analysis showed that the interaction between ZEB1 and miR-183-5p promoter DNA was reduced by AGGF1 because AGGF1 resulted in reduced ZEB1 levels (Fig. 6a). The data suggest that AGGF1 negatively regulates the level of ZEB1.

We and others have shown that AGGF1 inhibits ERK signaling^44, 49^, and a study in MCF-10A mammary epithelial cells showed that ERK regulates the level of *ZEB1* ^50^. Thus, we tested the hypothesis that AGGF1 regulates the level of *ZEB1* by regulating the ERK signaling pathway in H9C2 cells. Western blot analysis showed that AGGF1 protein treatment significantly reduced the phosphorylation level of ERK1/2 compared with control PBS treatment (Fig. 6f). The quantitative RT-PCR analysis showed that reduced *ERK1/2* expression by siRNA significantly increased the level of miR-183-5p (Supplementary Fig. 13). Western blot analysis showed that reduced *ERK1/2* expression by siERK1/2 significantly decreased the level of CHOP (Fig. 6h). Reduced ERK1/2 expression by siRNA significantly reduced the level of ZEB1 (Fig. 6g, h). These data were confirmed in stressed cells treated with ISO. Western blot analysis showed that AGGF1 treatment significantly decreased the levels of ZEB1 and phosphorylated ERK1/2 in H9C2 cells treated with ISO for 48 h (Supplementary Fig. 14). Together, our data indicate that AGGF1 negatively regulates activation of ERK1/2, which leads to a reduced level of *ZEB1*, resulting in an increased level of miR-183-5p. Increased miR-183-5p expression will reduce the level of *CHOP*, which blocks ER stress-induced apoptosis and heart failure. Moreover, AGGF1 increases the levels of p-eIF2a and ATF4 through inhibition of CHOP expression and GADD34 expression (Fig. 6i).

### MiR-183-5p blocks ER stress-induced apoptosis

**Fig. 7:**
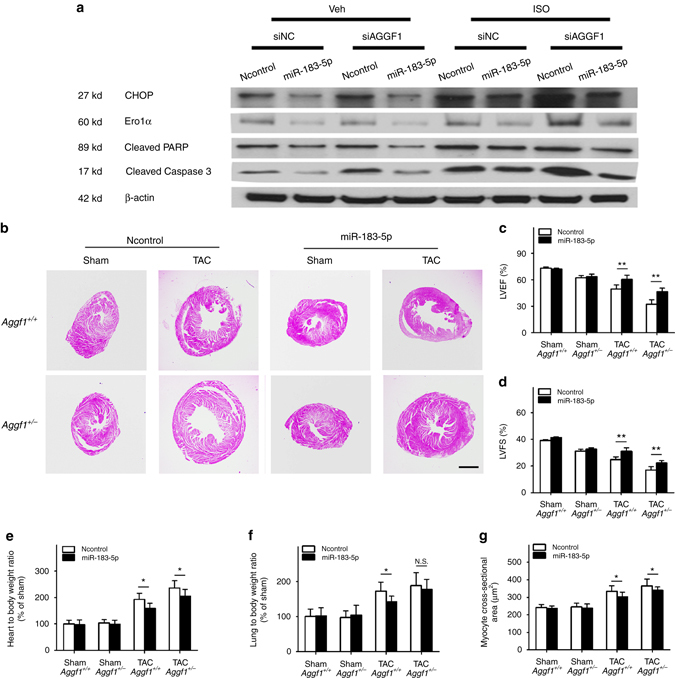
AGGF1 regulates ER stress and hypertrophy upstream of miR-183-5p. **a** Representative western blot images for ER stress signaling and apoptotic markers in H9C2 cells treated with ISO to induce hypertrophy or control vehicle (Veh) as well as siAGGF1 or siNC. The experiment was repeated three times. **b**–**g** Ago-miR-183-5p inhibits cardiac hypertrophy and improves cardiac function. **b** H&E staining images of heart sections. *Scale bar*, 1 mm. **c** LVEF. **d** LVFS. **e** Ratio of heart weight to body weight. **f** Ratio of lung weight to body weight. **g** Cross-sectional diameter (µm) of cardiomyocytes (*n* = 10/group, *P < 0.05, **P < 0.01, *N.S.*, Non-significant). Data are shown as the mean ± s.d. from at least three independent experiments. Statistical analysis was carried out by a Student’s two-tailed *t*-test

Because CHOP is the key regulator of ER stress and its expression is regulated by miR-183-5p, we assessed the direct role of miR-183-5p on ER stress-induced apoptosis. The ER stress activator TM increased the abundance of CHOP, ERO1α, and DR5, which was suppressed by miR-183-5p compared with Ncontrol (Supplementary Fig. 15). H9C2 cells were transfected with *Aggf1* siRNA vs. siNC together with miR-185-5p mimics or Ncontrol, and then treated with ISO or vehicle. Western blot analysis showed that miR-183-5p reduced the abundance of CHOP, ERO1α, cleaved PARP, and cleaved caspase-3 in the Veh-siNC group (Fig. 7a). This effect of miR-183-5p on ER stress-induced apoptosis was not affected by siAGGF1 (Fig. 7a, compare the Veh-siAGGF1 group to the Veh-siNC group). Similar observations were made in H9C2 cells treated with ISO (Fig. 7a, *panel ISO*). These data are consistent with our results that AGGF1 acts upstream of miR-183-5p in regulation of CHOP expression.

### MiR-183-5p mimics inhibits cardiac hypertrophy

To establish the role of miR-183-5p in cardiac hypertrophy and heart failure and to explore the relationship between miR-183-5p and AGGF1 during the process, we performed TAC procedures for both WT and *Aggf1* ^*+/−*^ mice, which were followed by intramuscular injection of Ago-miR-183-5p and control Ago-miR-NC. H&E staining and echocardiography showed that there is an additive effect for *Aggf1* haploinsufficiency and TAC in inducing cardiac hypertrophy and heart failure, which was rescued by Ago-miR-183-5p (Fig. 7b–g). Echocardiography showed that LVEF and LVFS were decreased in *Aggf1* ^*+/−*^ mice compared with WT mice; TAC reduced LVEF and LVFS in WT mice; Combination of TAC and *Aggf1* haploinsufficiency further aggravated hypertrophy and heart failure by reducing LVEF and LVFS more; Ago-miR-183-5p rescued the reduction of LVEF and LVFS induced by TAC in WT mice; Ago-miR-183-5p rescued the reduction of LVEF and LVFS induced by TAC and *Aggf1* haploinsufficiency (Fig. 7b–g). The finding that miR-183-5p can rescue the effect of *Aggf1* haploinsufficiency suggests that *Aggf1* acts upstream of miR-183-5p. The same conclusion can be made with regard to the HW/BW ratio (Fig. 7e), LW/BW ratio (Fig. 7f), and the cross-sectional diameter of cardiomyocytes (Fig. 7f). Ago-miR-183-5p decreased the HW/BW ratio (Fig. 7e) and the LW/BW ratio (Fig. 7f) in both *Aggf1* ^*+/−*^ mice and WT mice after TAC. The cross-sectional diameter of cardiomyocytes was decreased in the Ago-miR-183-5p treatment groups (Fig. 7g). These data suggest that miR-183-5p inhibits cardiac hypertrophy and improves myocardial function.

### MiR-183-5p inhibitor induces cardiac hypertrophy

**Fig. 8:**
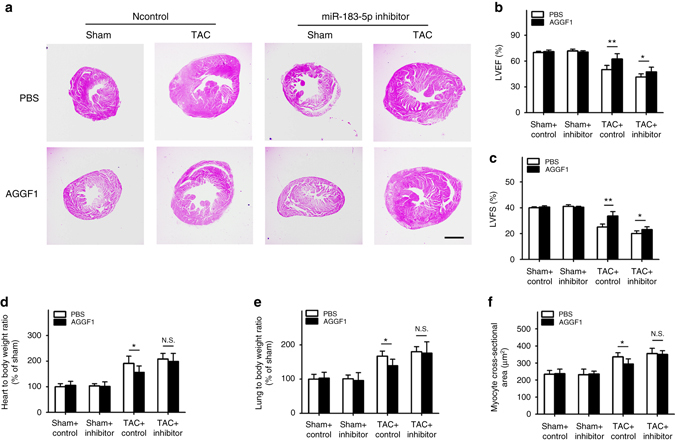
Ago-miR-183-5p inhibitor blocks therapeutic effect of AGGF1. **a** H&E staining images of heart sections. *Scale bar*, 1 mm. **b** LVEF. **c** LVFS. **d** Ratio of heart weight to body weight. **e** Ratio of lung weight to body weight. **f** Cross-sectional diameter (µm) of cardiomyocytes (*n* = 10/group, *P < 0.05, **P < 0.01, *N.S.*, Non-significant). Data are shown as the mean ± s.d. from at least three independent experiments. Statistical analysis was carried out by a Student’s two-tailed *t*-test

To test the hypothesis that miR-183-5p is involved in AGGF1-mediated treatment of hypertrophy and heart failure after TAC, we assessed the effect of AGGF1 protein treatment with or without an Antago-miR-183-5p inhibitor in mice after TAC. H&E staining showed that AGGF1 therapy reduced cardiac hypertrophy and heart failure in 6-week-old TAC mice, but the therapeutic effect was lost in the presence of the Antago-miR-183-5p inhibitor (Fig. 8a). Similarly, echocardiography showed that AGGF1 increased LVEF and LVFS in TAC mice, but the effect was abolished in the presence of the Antago-miR-183-5p inhibitor (Fig. 8b, c). AGGF1 rescued the HW/BW ratio and the LW/BW ratio in TAC mice, but the effect was abolished in the presence of the Antago-miR-183-5p inhibitor (Fig. 8d, e). AGGF1 reduced the cross-sectional diameter of cardiomyocytes in TAC mice, but the reduction was abolished in the presence of the Antago-miR-183-5p inhibitor (Fig. 8f). All these data suggest that the therapeutic effect of AGGF1 protein is dependent on miR-183-5p and AGGF1 acts upstream of miR-183-5p.

## Discussion

The present study has identified a novel in vivo physiological role of AGGF1 in regulating ER stress signaling, and blocking ER stress-induced apoptosis, cardiac hypertrophy, and heart failure. We used *Aggf1* ^*+/−*^ mice to explore the in vivo role of *Aggf1* in the heart as homozygous *Aggf1*KO mice were embryonically lethal. We found that *Aggf1* had an important pathophysiological role in the development of cardiac hypertrophy and heart failure. Pressure overload by TAC induced cardiac hypertrophy and heart failure, which was exacerbated significantly in *Aggf1* ^*+/−*^ mice (Fig. 1). Western blot analysis showed that *Aggf1* haploinsufficiency significantly regulated TAC-induced ER stress signaling in cardiac tissue samples. Moreover, direct injection of AGGF1 protein significantly reduced TAC-induced cardiac apoptosis, hypertrophy, and heart failure, and restored myocardial function in TAC mice to a nearly normal level. AGGF1 protein treatment also regulated ER stress signaling and blocked ER stress-induced apoptosis in an in vitro ISO-induced H9C2 cell model for hypertrophy. These data suggest that AGGF1 is an important molecular determinant for ER stress signaling, cardiac hypertrophy, and heart failure.

During ER stress signaling, one key feature is translational attenuation by phosphorylation of the translation initiation factor eIF2a by a ER stress sensor, PERK, which prevents translation of most proteins, but allows translation of only specific mRNAs, such as *ATF4* and *CHOP* ^29, 33^. Translation of ATF4 activates the expression of CHOP by direct binding to the 5′-UTR region of *CHOP* ^29^. CHOP can then activate further downstream genes during ER stress, leading to induction of cell death by apoptosis. In this study, we found that AGGF1 regulated the abundance of ER stress signaling markers, including sXBP1, Bip, CHOP, ERO1α, and DR5; however, the levels of p-eIF2a and ATF4 were increased after AGGF1 treatment due to decreased GADD34 expression (Fig. 4). Moreover, TAC-induced ATF4 expression was abolished in heterozygous *Aggf1* ^*+/−*^ mice (Fig. 1). Therefore, AGGF1 regulates ER stress signaling by a non-canonical CHOP pathway (Fig. 6). We have found that AGGF1 induces expression of miR-183-5p, which then reduces the CHOP expression by a post-transcriptional suppression mechanism through binding to the 3′-UTR of *CHOP* (Fig. 5). We further demonstrated that AGGF1 negatively regulates activation of ERK1/2, leading to a decreased level of transcriptional repressor ZEB1, resulting in increased expression of miR-183-5p (Fig. 6). Therefore, our data identify a novel non-canonical CHOP pathway for ER stress signaling, i.e., AGGF1-ERK-ZEB1-miR-183-CHOP (Fig. 6i). However, it remains to be established how AGGF1 regulates ERK1/2 activation in the future.

Enhanced phosphorylation of eIF2α reduces protein translation and has been reported to mediate the inhibition of protein synthesis. Fu et al. argued that increased phosphorylation of eIF2α led to translational inhibition in *Chop*-deficient mice, thereby inhibiting cardiac hypertrophy in these mice^24^. Moreover, a *Chop* deficiency led to decreased apoptosis, also inhibiting heart failure. We found that AGGF1 increased the phosphorylation of eIF2a through negative feedback reduction of GADD34 by reduced CHOP abundance (Fig. 4). Increased phosphorylation of eIF2α may be one of the major factors contributing to the suppression of cardiac hypertrophy by AGGF1 through the inhibition of protein synthesis. Furthermore, a high level of CHOP leads to cardiomyocytes’ apoptosis by a decreased level of Bcl2 and an increased level of Bax. AGGF1 protein therapy inhibited heart failure and restored myocardial function (LVEF and LVFS) to nearly normal levels by blocking CHOP-induced apoptosis (Fig. 3).

Left ventricular hypertrophy (LVH) is a high-risk factor for cardiovascular mortality. If untreated or delayed, LVH leads to cardiac dysfunction and finally induces heart failure. Although clinical treatment of cardiac hypertrophy and heart failure advanced substantially, the current treatment options are limited, transplantation is often necessary, and high mortality remains. Therefore, the development of novel treatments is critical. It is of high interest that AGGF1 protein therapy inhibited cardiac hypertrophy and heart failure and restored myocardial function (LVEF and LVFS) to nearly normal levels by blocking ER stress-induced apoptosis (Figs. 3 and 4). Moreover, miR-183-5p also inhibited myocardial apoptosis, reduced cardiac hypertrophy and heart failure, and restored myocardial function (Fig. 7). The studies with the miR-183-5p inhibitor showed that AGGF1 acts upstream of miR-183-5p in regulating cardiac hypertrophy and heart failure (Fig. 8). Mechanistically, we showed that AGGF1 blocked ERK1/2 activation, repressed expression of ZEB1, and induced expression of miR-183-5p, which then inhibited expression of CHOP, the key inducer of apoptosis. It is important to note that miR-183-5p is the first microRNA identified for post-transcriptional regulation of CHOP. It is interesting to note that the Ago-miR-183-5p mimics and Antagomir-miR-183-5p inhibitor also have a major impact on cardiac hypertrophy and dysfunction upon pressure overload regardless of *Aggf1* expression or AGGF1 protein treatment, respectively (Figs. 7 and 8). Therefore, miR-183-5p by itself may have an impact on cardiac pathology independent from AGGF1. Our study suggests new treatment targets for cardiac hypertrophy and heart failure.

In addition to cardiac hypertrophy and heart failure, recent animal and human studies have shown that ER stress is associated with other cardiovascular diseases, including atherosclerosis and plaque rupture, ischemic heart disease, hypertension, and cardiotoxicity of anticancer drug treatments^21^. There is a possibility that AGGF1 protein therapy may be an attractive therapeutic intervention for these pathological cardiovascular processes. Moreover, ER stress is an evolutionarily conserved process involved in numerous other non-cardiovascular diseases^22^. Thus, AGGF1 protein therapy that regulates ER stress signaling may also be a promising strategy to clinically intervene with these non-cardiovascular diseases.

Because cardiac function was only partially rescued in *Chop* KO mice in a TAC model^24^, whereas AGGF1 protein therapy almost completely restored myocardial function, the AGGF1-ERK-ZEB1-miR-183-CHOP signaling pathway may be one of several pathways by which AGGF1 restores myocardial function. Recently, we have found that AGGF1 protein induces autophagy in HL1 and H9C2 cells and mouse hearts^45^. AGGF1-induced autophagy can protect cardiomyocytes from apoptosis and inhibit fibrosis, thereby improving myocardial function. The combined effect of AGGF1-induced autophagy and AGGF1-mediated ER stress signaling may be the key for the successful inhibition of cardiac hypertrophy and heart failure and increased myocardial function. The detailed relationship between AGGF1-regulated autophagy and ER stress signaling remains an interesting topic to be investigated in the future. Furthermore, we have reported that AGGF1 promotes angiogenesis and inhibits vascular permeability in the heart^45^, whereas angiogenesis and vascular permeability are impaired in heterozygous *Aggf1* ^*+/−*^ hearts^44^. Interestingly, the AGGF1 protein therapy increased the ventricular angiogenesis in TAC mice treated with the AGGF1 protein than those treated with control PBS or sham mice (Supplementary Fig. 5). Coronary angiogenesis was reported to be increased at the initial stage of pathological cardiac hypertrophy, but reduced at the late stage^51–53^. Therefore, it is possible that reduced angiogenesis and increased vascular permeability in *Aggf1* ^*+/−*^ mice may contribute to exacerbation of pathological cardiac hypertrophy in these mice. On the other hand, increased angiogenesis by the AGGF1 protein therapy may suppress cardiac hypertrophy and heart failure in TAC mice.

In summary, we show that AGGF1 haploinsufficiency affects ER stress signaling and causes cardiac hypertrophy and heart failure. On the other hand, AGGF1 protein therapy inhibits cardiac hypertrophy and heart failure by blocking ER stress-induced cardiomyocyte apoptosis. AGGF1 regulates ER stress signaling by a novel AGGF1-ERK-ZEB1-miR-183-CHOP signaling pathway. Our studies in mouse models have indicated that AGGF1 protein therapy is a novel treatment strategy for cardiac hypertrophy and heart failure. As ER stress is an evolutionarily conserved process involved in numerous diseases, AGGF1 protein therapy may serve as a new treatment not only for cardiovascular diseases, but also for many other diseases associated with ER stress.

## Methods

### Antibodies

Antibodies for KDEL receptor (sc-33806, 1:50 dilution for immunostaining), Bax (sc-493, 1:500 dilution for western blot), and Bcl2 (sc-7382, 1:1000 dilution for western blot) were from Santa Cruz Biotechnology (Santa Cruz, CA, USA). Antibodies for CHOP (#2895, 1:1000 dilution for western blot), cleaved caspase-3 (#9662, 1:1000 dilution for western blot), Ero1α (#3264, 1:1000 dilution for western blot), sXBP1 (#12782, 1:1000 dilution for western blot), and cleaved PARP (#9542, 1:1000 dilution for western blot) were from Cell Signaling Technology (Boston, MA, USA). Antibodies for BiP (ab21685, 1:500 dilution for western blot), cATF6 (ab11909, 1:1000 dilution for western blot), and GAPDH (ab9484, 1:1000 dilution for western blot) were from Abcam (Cambridge, MA, USA). Antibodies for AGGF1 (11889-1-AP, 1:1000 dilution for western blot), ATF4 (10835-1-AP, 1:500 dilution for western blot), β-actin (60008-1-Ig, 1:1000 dilution for western blot), DR5 (15497-1-AP, 1:500 dilution for western blot), and Puma (55120-1-AP, 1:500 dilution for western blot) were from Proteintech (Wuhan, Hubei, China). An antibody for p-PERK (DF7576, 1:1000 dilution for western blot) was from Affinity Biosciences (Cincinnati, OH, USA), and an antibody for p-eIF2α (1:200 dilution for western blot) was from Bioss (Beijing, China).

### siRNAs and miRNA reagents

siRNAs were purchased from Guangzhou RioboBio (Guangzhou, Guangdong, China). The sequence for an *AGGF1*-specific siRNA is 5′-GGAGUCUCCUGGAGAUGAUTT-3′, whereas the sequence of the Ncontrol siRNA (siNC) is 5′-UUCUCCGAACGUGUCACGUTT-3′, which does not match any human gene.

MiR-183-5p mimics, Ncontrol miRNA mimics (Ncontrol), a miR-183-5p inhibitor, a miR-183-5p inhibitor control, and stabilized miRNAs (Ago-miR-183-5p and control Ago-miR-NC) were from Guangzhou RioboBio.

### Plasmids

The whole 3′-UTR of the *DDIT3* gene encoding CHOP (216 bp) was amplified by PCR using human genomic DNA and primers 5-AGCTACGCGTACAATTGGGAGCATCAGTCCCC-3′ (forward) and 5′-AGCTAAGCTTTGGCTCATAGAAAGTCACTTTAATAGATAGG-3′ (reverse). The PCR product was digested with *Sac I* and *Hind III* restriction enzymes and subcloned into the vector pMIR-REPORT luciferase (Applied Biosystems, Foster City, CA, USA) cut with the same enzymes. This created a luciferase reporter referred to as pMIR-CHOP-wt. The miR-183-5p-binding site at the 3′-UTR of *CHOP* was mutated in the pMIR-CHOP-wt reporter using PCR-based site-directed mutagenesis as described previously^54^, resulting in a mutant reporter, pMIR-CHOP-mut.

### Cell culture and transfection

H9C2 rat cardiomyoblasts were obtained from ATCC (Rockville, MD, USA; CRL-1446™), cultured in the Dulbecco’s Modified Eagle’s medium (DMEM) supplemented with 15% fetal bovine serum (FBS) in a humidified incubator with 5% CO_2_ at 37 °C. H9C2 cells were transfected with siRNA, miRNA mimics, and miRNA inhibitors using Lipofectamine 2000 according to the manufacturer’s instructions (Invitrogen, Carlsbad, CA, USA). Cells were discarded if contaminated by mycoplasma and other microorganisms.

### Dual luciferase assays

H9C2 rat cardiomyoblasts were cultured in 24-well plates. After 24 h, we co-transfected 200 ng of either pMIR-CHOP-wt or pMIR-CHOP-mut together with 100 nM of miR-183-5p mimics or non-target miRNA mimics as well as 20 ng of the pRL-TK vector containing the renilla luciferase gene (Promega, Madison, WI, USA) using Lipofectamine 2000. Forty-eight hours after transfection, cells were harvested, lysed using 1× passive lysis buffer (Promega) and used for luciferase assays according to the manufacturer’s instruction (Gibco Life Technologies, Gaithersburg, MD, USA). Firefly and renilla luciferase activities were measured using the Dual-Glo luciferase assay kit (Gibco Life Technologies). The experiments were repeated at least three times.

For luciferase assays for the miR-183-5p promoter-luciferase reporter, the pGL3-miR-183 reporter or control pGL3 was co-transfected with pRL-TK, and luciferase activities were measured 48 h later using the Dual-Glo luciferase assay kit (Gibco Life Technologies) as described above.

### Tissue samples of human hearts

Human heart samples were obtained from eight patients with dilated cardiomyopathy undergoing cardiac transplantation and three control heart samples from unmatched or rejected healthy donor hearts. The human subject studies were approved and performed according to the standards established by the Ethics Committee on Human Subject Research at Huazhong University of Science and Technology and by the Cleveland Clinic Foundation Institutional Review Board on Human Subjects. This study conformed to the guidelines set forth by the Declaration of Helsinki, and all participants have provided written informed consent.

### Animals and procedures

Male C57BL/6 mice (12 weeks) were used for all studies. The *Aggf1* ^*+/−*^ mice were generated in our laboratory using a gene-trap methodology^44^. Animal care and experimental procedures were approved by the Ethics Committee on Animal Research of Huazhong University of Science and Technology and the Institutional Animal Care and Use Committee of Cleveland Clinic.

Pressure overload of the heart was induced in 12-week-old male mice (20–25 g) by TAC. Mice were anesthetized with an intraperitoneal injection of sodium pentobarbital (50 mg/kg). The chest was opened via minithoracotomy to expose the aortic arch, and TAC was performed by tying a 7–0 silk suture around a 27-gauge needle overlying the arch at the point between the brachiocephalic trunk and left common carotid artery. Animals were randomly assigned to different study groups (*n* = 12). The sample size was determined using the nQuery Advisor 7.0 software with assumptions of a power of 80%, a *P* value of 0.01, and an effect size of 1.5–2. Animals that did not survive the surgeries were excluded from further analysis.

ALZET Model 1004 minipumps (Cupertino, CA, USA) were implanted intraperitoneally to administer ISO to mice at a dose of 30 mg/kg BW/day for 28 days.

AgomiR-183-5p (100 nmol/kg), AntagomiR-NC (100 nmol/kg), a miR-183-5p inhibitor (200 nmol/kg), and a miR-183-5p inhibitor control (200 nmol/kg) were administered by intramuscular injection into the left ventricle myocardium at multiple sites in 0.2 ml of saline 24 h prior to TAC. The dosage of AgomiR was determined according to the published studies^55^.

For protein therapy, 2 weeks after the surgery, the mice were injected intravenously with AGGF1 (0.25 mg/kg BW) or the same dose of PBS twice a week for 4 weeks.

To overexpress *AGGF1* in mice, C57BL/6 mice were anesthetized and injected with 0.2 ml of adenovirus (AAV9-AGGF1 viruses or control AAV9-GFP viruses; Vector Biolabs) into the left ventricular myocardium using a 35-gauge needle at multiple sites.

Echocardiography was performed with a Vevo2100 High-Resolution Micro-Ultrasound System (Visual Sonics, Toronto, Canada) at four different study time points: pre-surgery, 2 weeks, 4 weeks, and 6 weeks after the surgery. Echocardiography was performed by an operator who was blinded to treatments.

We measured the blood pressure of mice using the tail-cuff method (BP-98a, Japan) after 7 days of training sessions. We placed the mice in a dark chamber at 37 °C for 30 min and then transferred them to a dark cage with a heating pad. We then monitored the tail-cuff pressure and recorded the signals using tail-cuff plethysmography (BP-98A; Softron Co., Tokyo, Japan). The SBP was calculated from 20 readings for each mouse. The SBP measurements were performed by one person who was blinded to the treatment groups.

### Histochemical analysis

Hearts were excised from mice, embedded in paraffin, sectioned into 4 μm slices, and stained with H&E^56^. To determine the expression level of ER stress chaperons, immunohistochemical staining was performed on paraffin-embedded sections with a primary antibody against the KDEL receptor (1:50 dilution), and then with a biotinylated secondary antibody (undiluted goat anti-rabbit/mouse (H+L), Dako Denmark A/S, Hovedstaden, Denmark)^56^. The sections were then treated with peroxidase-conjugated biotin–avid in complex using VECTASTAIN ABC-AP and visualized by DAB (3, 3-diaminobenzidine).

### Quantitative real-time RT-PCR analysis

Total RNA was extracted from cultured cells or mouse hearts using Trizol (Invitrogen) according to the manufacturer’s instruction. A total of 0.5 μg of RNA samples was reverse-transcribed using M-MLV reverse transcriptase according to the manufacturer’s protocol (Promega). Quantitative real-time PCR analysis was then performed using the FastStart Universal SYBR Green Master (Roche, Basel, BS, Switzerland) and a 7900 HT Fast Real-Time PCR System (Thermo, Waltham, MA, USA) as described previously^40^. Experiments were performed in triplicate and repeated at least three times.

### Western blot analysis

Western blot analysis was carried out using different cell extracts and heart samples with different antibodies. Mice were anesthetized with an intraperitoneal injection of sodium pentobarbital (50 mg/kg) and hearts were dissected out for extraction of total proteins. Cultured H9C2 was washed three times with PBS and then used for extraction of total proteins. Protein extracts were prepared by lysis in 20 mM Tris-HCl, pH 7.6, 150 mM NaCl, 0.1% DOC, 0.5% NP-40, 10% glycerol, 1 mM glycerophosphate, 1 mM NaF, 2.5 mM Na pyrophosphate, 1 mM Na_3_VO_4_, and a cocktail of protease inhibitors (Calbiochem) at 4 °C. Protein extracts were mixed with the reducing laemmLi sample buffer, boiled for 12 min, separated by SDS-polyacrylamide gel electrophoresis, and transferred to nitrocellulose membranes. The membranes were cut into two or more horizontal strips from the top to the bottom based on the predicated molecular weights of the target protein(s) and controls, and then blotted individually with appropriate primary antibodies and appropriate secondary antibodies. Images from western blot analysis were captured and quantified using 1-D Analysis Software and Quantity One (Bio-Rad, Hercules, CA, USA). The experiments were repeated at least three times. Full-image scan results of western blots are shown in Supplementary Figs. 16–20.

### ChIP assays

We used ChIP coupled with quantitative PCR (qPCR) to characterize protein–DNA interaction. ChIP assays were performed as directed by us previously^57^ using a kit from Beyotime (Haimen, China). In brief, H9C2 cells were grown in a 10-cm dish. Chromatin was crosslinked with 1% formaldehyde. Cells (5 × 10^6^) were then lysed with 500 μl of ChIP lysis buffer (1% SDS, 10 mM EDTA, 50 mM Tris-HCl, pH 8.0) with a cocktail of protease inhibitors (Calbiochem) for 10 min on ice. The samples were sonicated to yield DNA fragments with an average size of ~200 bp. Protein–DNA complexes were immunoprecipitated with IgG or an anti-ZEB1 antibody (2 μg; Proteintech). The immunoprecipitated material was washed and heated at 65 °C overnight to reverse the crosslink. The ChIP DNA was column-purified (Qiagen, Valencia, CA, USA), and used for qPCR.

### Analysis of plasma ANF levels

Blood samples were collected from the hearts of mice. Plasma ANF levels were determined using an ELISA kit specific for ANF from Abcam. The experiments were repeated at least three times.

### Statistical analysis

All quantitative data were shown as mean ± s.d. The difference between two groups of variables was compared by the two-tailed, paired or unpaired Student’s *t*-test. For comparisons of more than two groups, one-way analysis of variance was employed for normal distributions and the Kruskal–Wallis test for non-normal or small samples. A *P* value of < 0.05 was considered as significant.

### Data availability

All data in the manuscript are available from the authors upon request.

## Electronic Supplementary Material

Supplementary Information
