# Supplementary material for: A non-canonical pathway regulates ER stress signaling and blocks ER stress-induced apoptosis and heart failure
Source: Nat Commun. 2017 Jul 25;8:133. doi: 10.1038/s41467-017-00171-w (PMC5527107; doi:10.1038/s41467-017-00171-w)
Supplement: Supplementary file 1 — Supplementary Information [file 41467_2017_171_MOESM1_ESM.pdf]

File Name: Supplementary Information
Description: Supplementary Figures and Supplementary Table.

Supplementary Figures

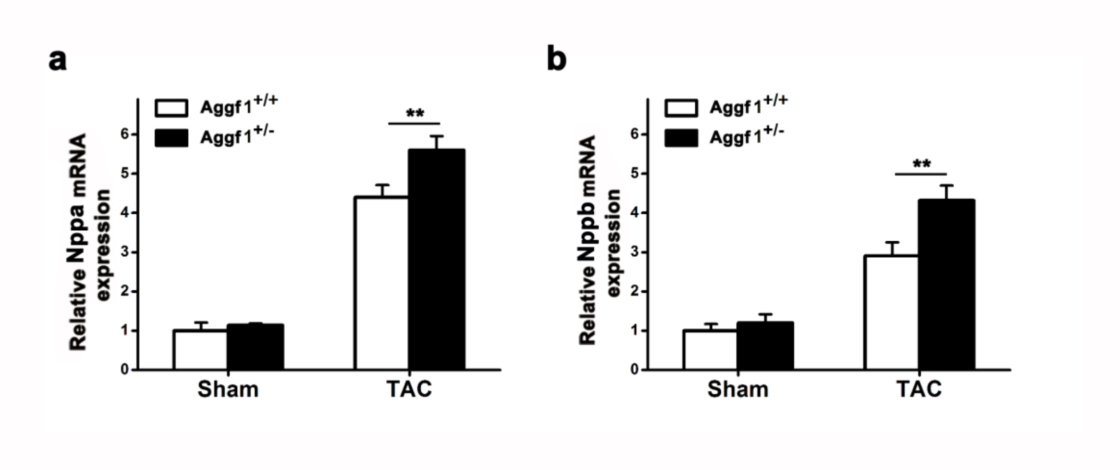

Supplementary Figure 1. Heterozygous *Aggf1* mice increases the expression levels of *Nppa* and *Nppb* after TAC.

Real time RT-PCR analysis was performed for *Nppa* encoding ANP **(a)** and *Nppb* encoding BNP **(b)** using total RNA samples isolated from *Aggf1*$^{+/-}$ mice or wild type *Aggf1*$^{+/+}$ mice with TAC or Sham surgeries (n=12/group, ***P*<0.01). Data are presented as mean ± s.d. from at least three independent experiments. Statistical analysis was carried out by a Student's two-tailed t-test.

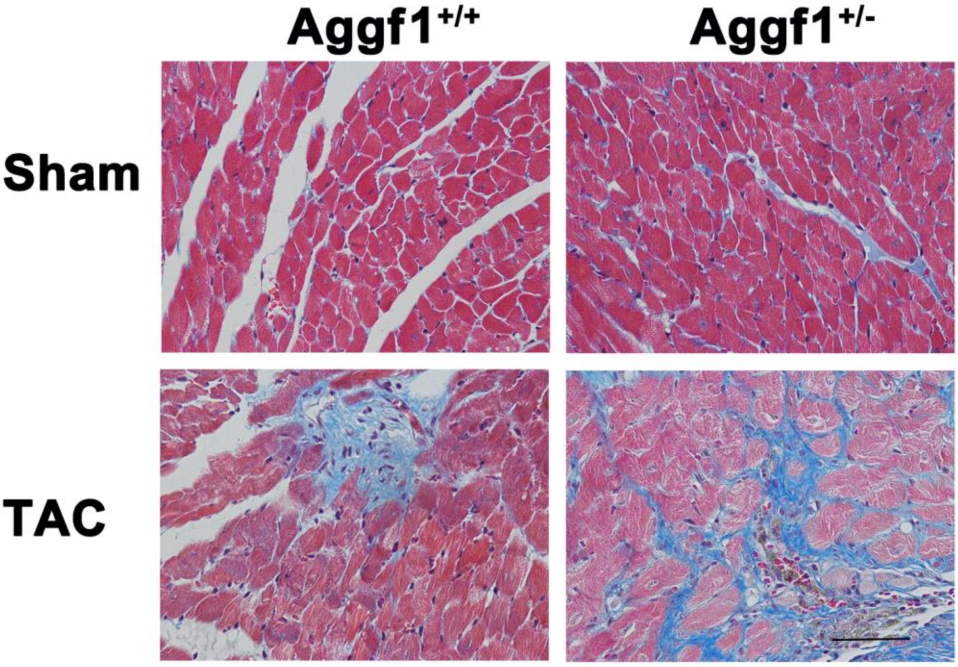

Supplementary Figure 2 ***Aggf1*** $^{+/-}$ **mice showed an enhanced level of myocardial fibrosis induced by TAC with Masson trichrome staining**. Scale bar, 50 μm.

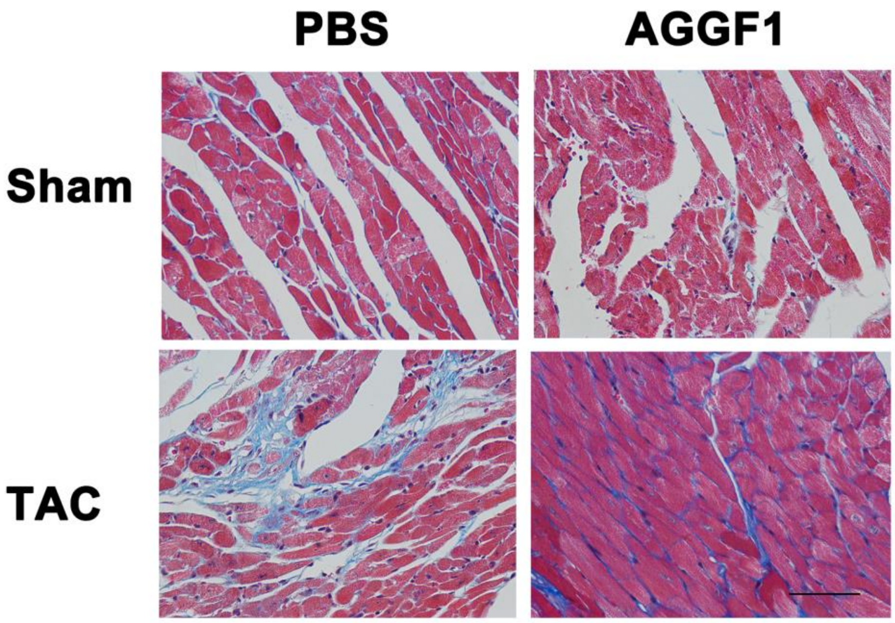

Supplementary Figure 3 The AGGF1 protein therapy inhibits myocardial fibrosis induced by TAC with Masson trichrome staining. Scale bar, 50 μm.

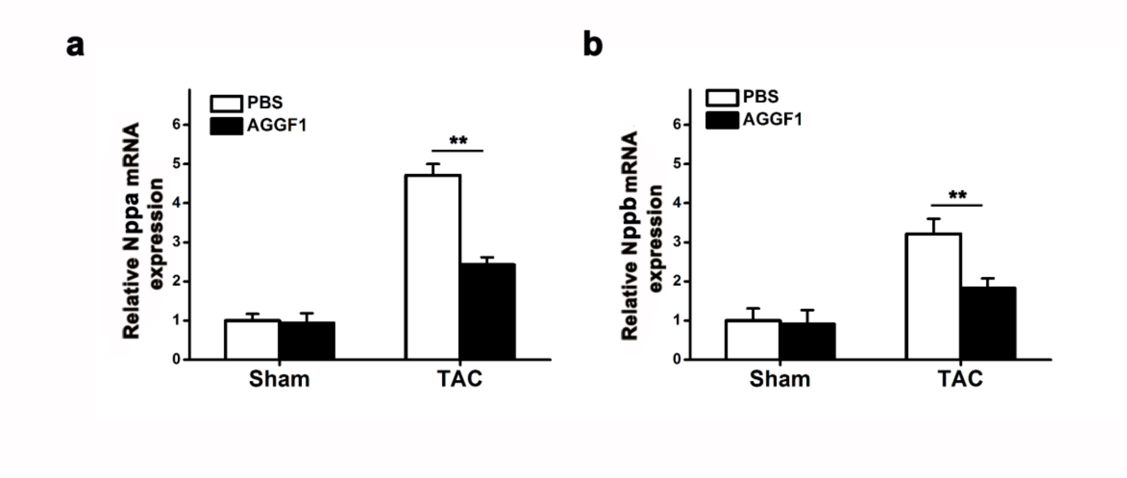

Supplementary Figure 4 The AGGF1 protein treatment significantly decreased the expression levels of *Nppa* (a) and *Nppb* (b) after TAC. Data are presented as mean ± s.d. from at least three independent experiments (n=12/group, $^{**}P<0.01$). Statistical analysis was carried out by a Student's two-tailed t-test.

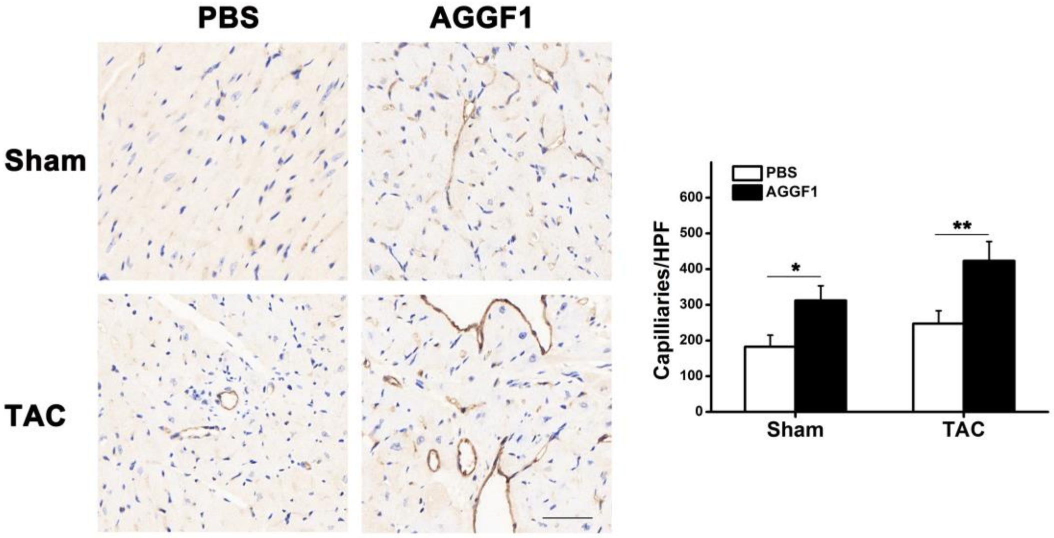

Supplementary Figure 5 The AGGF1 protein treatment increases the ventricular vascular density as shown by increased CD31-positive vessels in TAC and control sham mice. Scale bar, 50 μm. Data are presented as mean ± s.d. from at least three independent experiments (n=8/group, **P*<0.05, ***P*<0.01). Statistical analysis was carried out by a Student's two-tailed t-test.

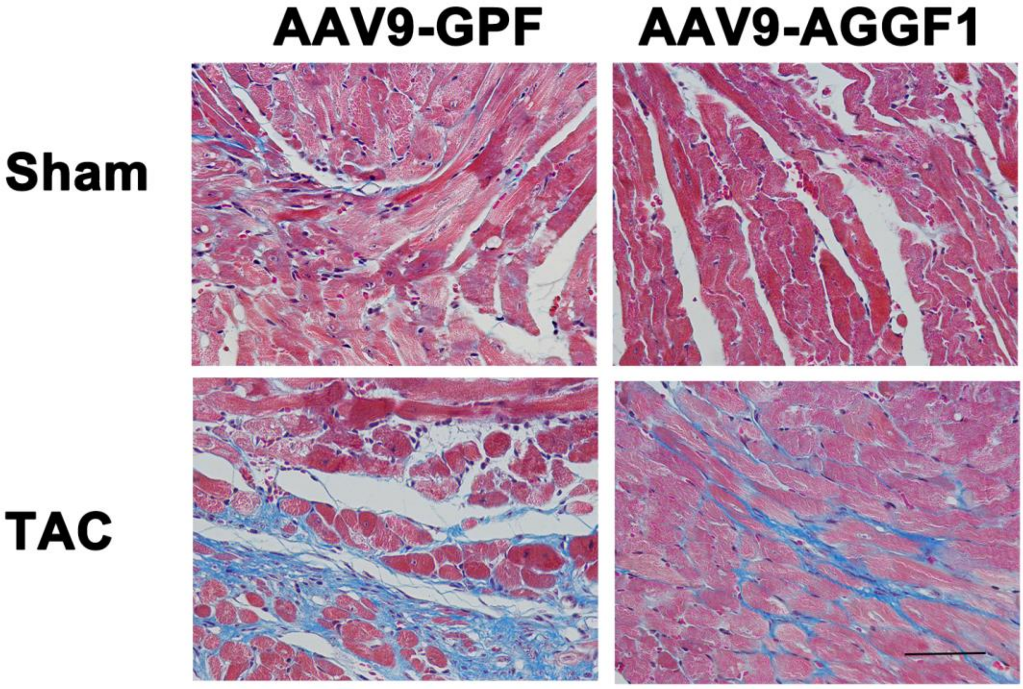

Supplementary Figure 6 Gene delivery by AAV9-AGGF1 viruses inhibits myocardial fibrosis induced by TAC with Masson trichrome staining. Scale bar, 50 μm.

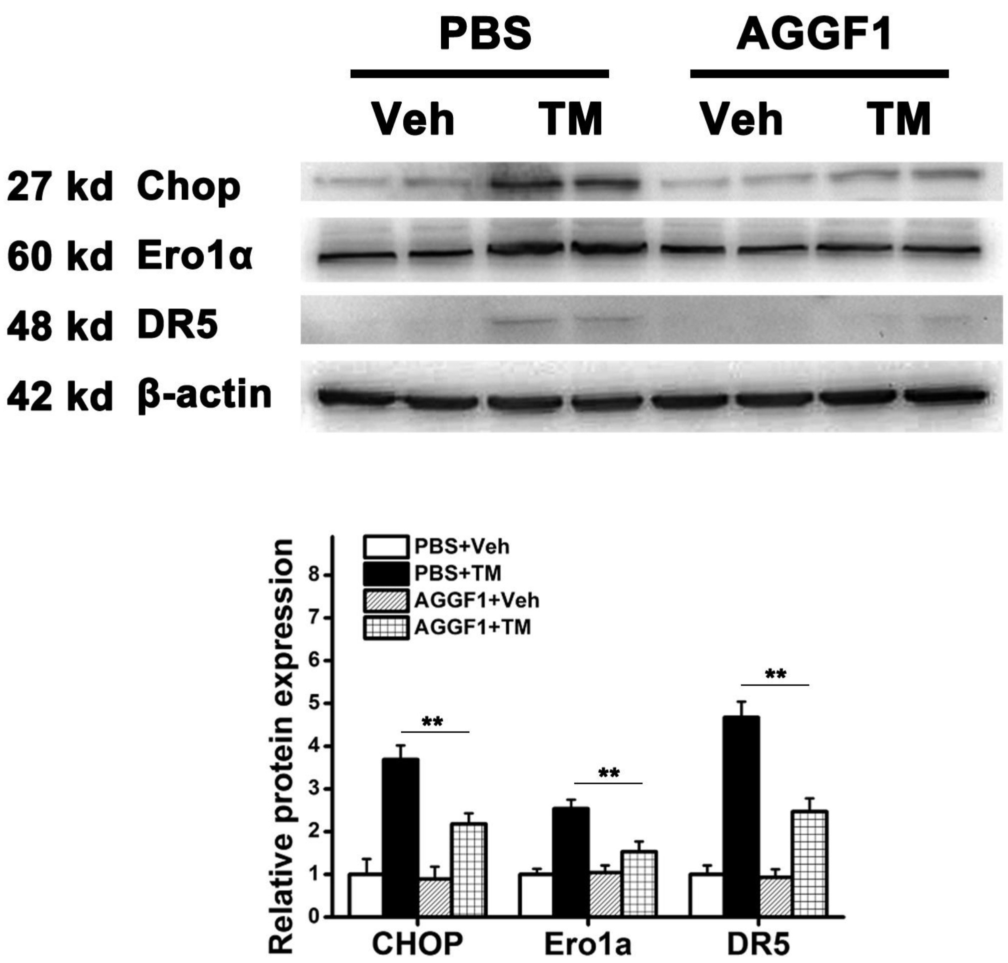

Supplementary Figure 7 The AGGF1 treatment reduces ER stress activator Tunicamycin (TM)-induced expression of CHOP, Ero1α, and DR5. Data are presented as mean ± s.d. from at least three independent experiments (n=6/group, ***P*<0.01). Statistical analysis was carried out by a Student's two-tailed t-test.

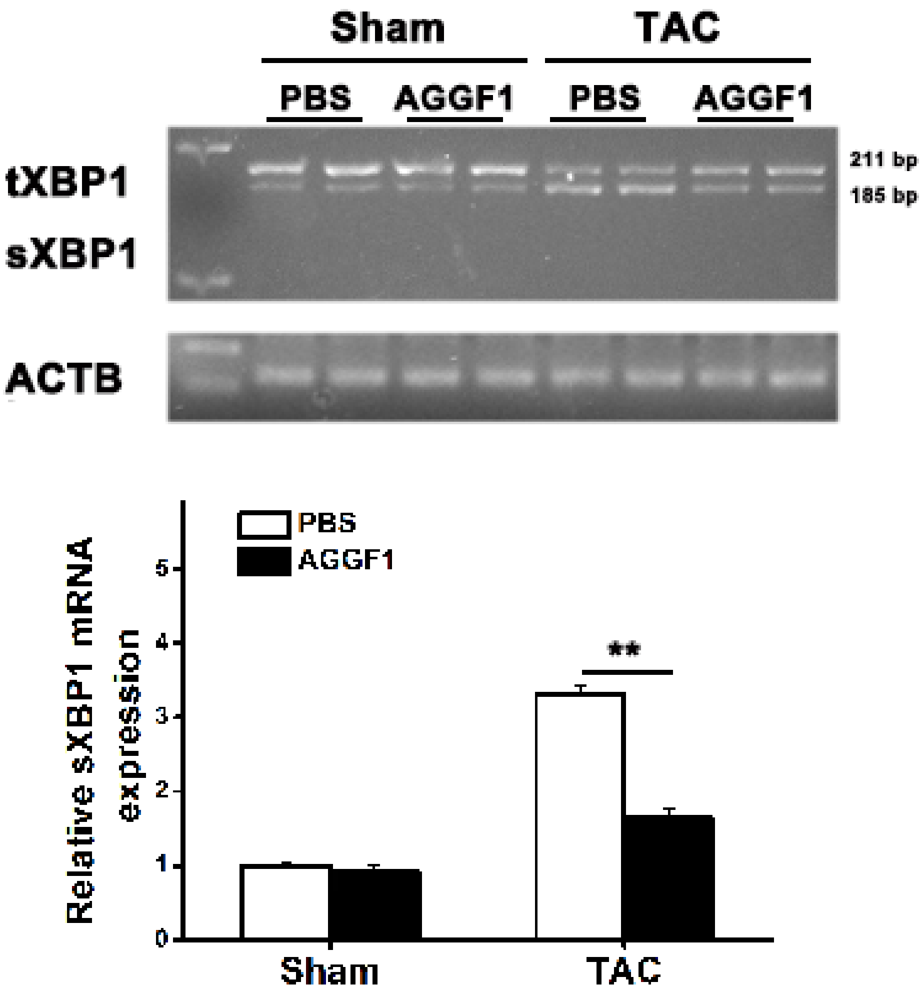

Supplementary Figure 8 Semi-quantitative RT-PCR analysis to characterize alternative splicing of *XBP1*. Total RNA samples were isolated from TAC mice or control sham mice treated with AGGF1 (0.25 mg/kg body weight) or control PBS, and reverse-transcribed into cDNA. PCR was then carried out using the cDNA and *XBP1* primers (Supplementary Table 1). The RT-PCR products were then separated by 3% agarose gels. Two PCR bands were detected. The 211 bp and 185 bp bands represent the alternatively spliced *XBP1* transcripts with or without the 26 nt intron 3. The *ACTB* (encoding β-actin) was used as loading control (123 bp). The intensity of the 185 bp spliced *XBP1* transcript (*sXBP1*) over the *ACTB* transcript was quantified and plotted at the bottom. (n=6/group, $^{**}P<0.01$). Data are shown as mean ± s.d. from at least three independent experiments. Statistical analysis was carried out by a Student's two-tailed t-test.

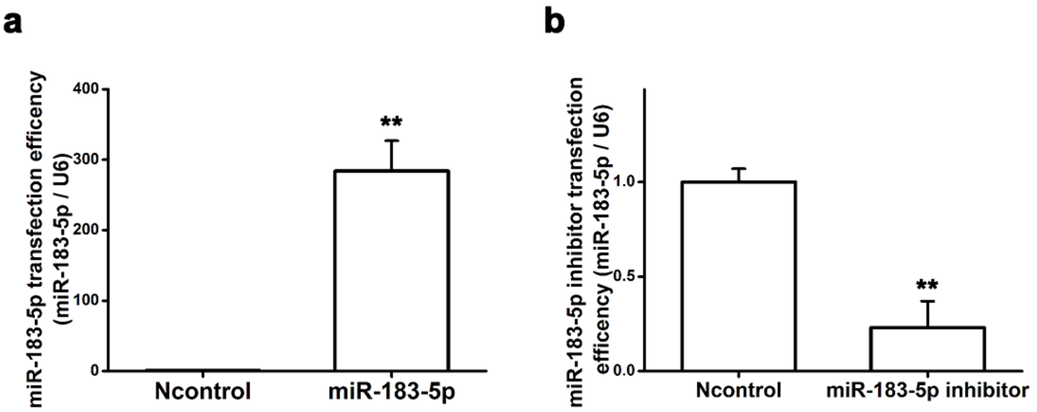

Supplementary Figure 9 Quantitative qPCR analysis of *miR-183-5p*.

(**a**) Transfection of the *miR-183-5p* mimics successfully increased the expression level of *miR-183-5p* in H9C2 cells compared with negative control (Ncontrol) (n=3/group, ***P*<0.01).

(b) Transfection of the *miR-183-5p* inhibitor successfully reduced the expression level of *miR-183-5p* in H9C2 cells compared with negative control (Ncontrol) (n=3/group, ***P*<0.01).

Data are shown as mean ± s.d. from at least three independent experiments. Statistical analysis was carried out by a Student's two-tailed t-test.

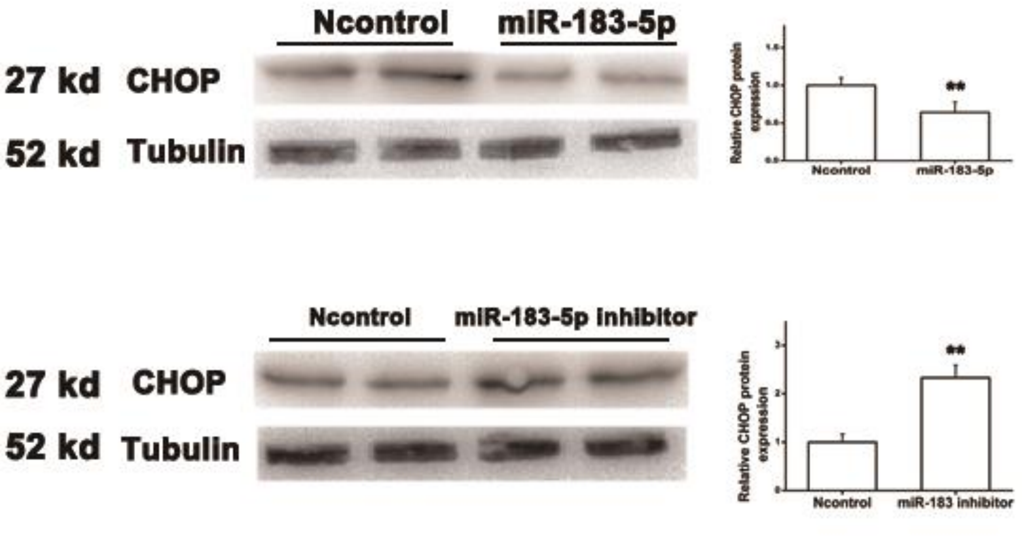

Supplementary Figure 10 Western blot analysis for CHOP expression in the hearts from TAC mice injected with Ago-miR-183-5p vs. Ago-miR-NC or Antago-miR-183-5p vs. Antago-miR-NC. Data are shown as mean ± s.d. from at least three independent experiments (n=6/group, $**P<0.01$). Statistical analysis was carried out by a Student's two-tailed t-test.

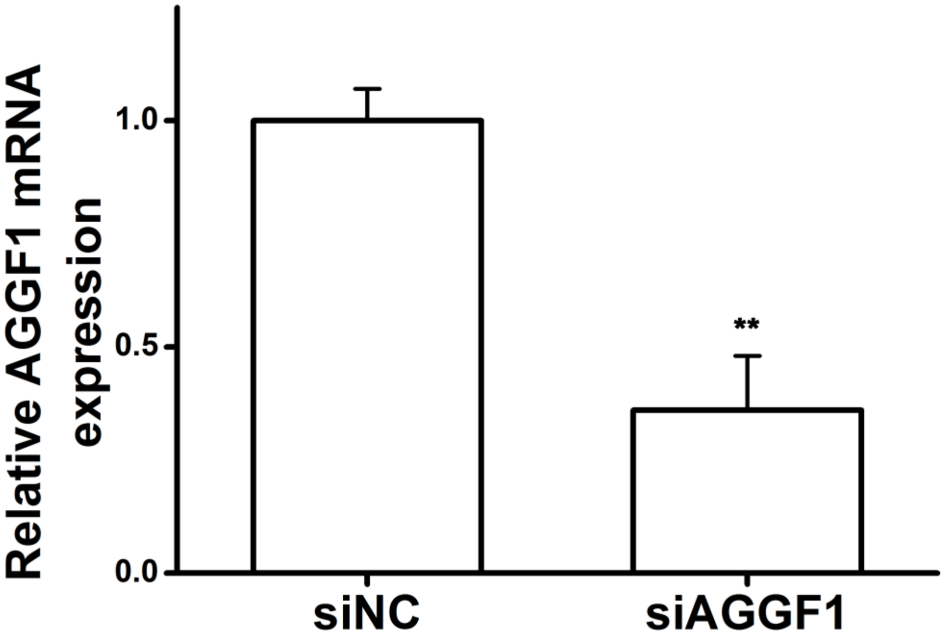

Supplementary Fig. 11 Real-time RT-PCR analysis for *AGGF1* expression in H9C2 cells by *AGGF1* siRNA (siAGGF1) vs. negative control scramble siRNA (siNC). Data are presented as mean ± s.d. from at least three independent experiments (n=3/group, $^{**}P<0.01$). Statistical analysis was carried out by a Student's two-tailed t-test.

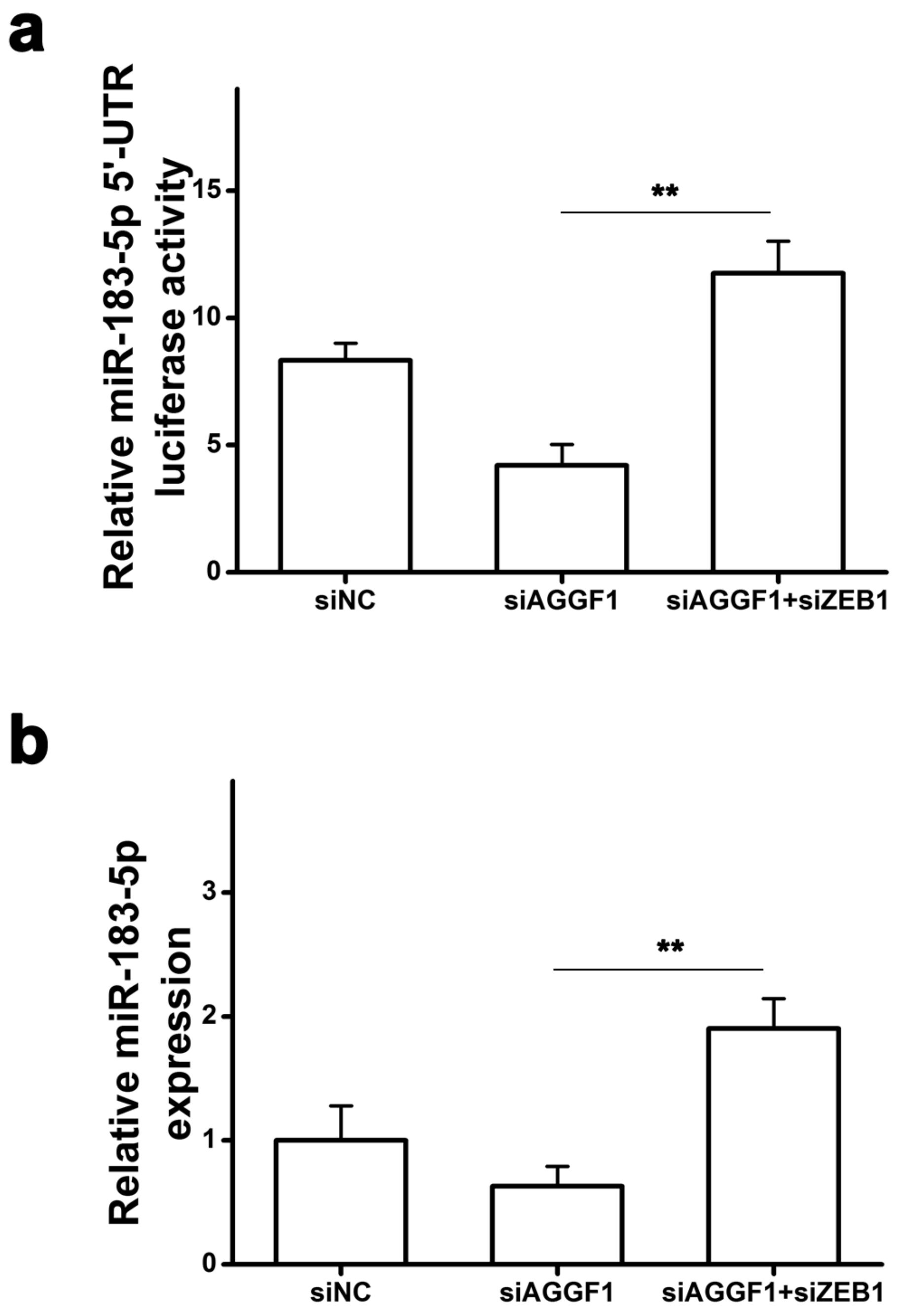

Supplementary Fig 12 ***AGGF1*** **acts upstream of** ***ZEB1*** **in the regulation of** ***miR-183-5p*** **expression.**

(a) Relative *miR-183-5p* promoter luciferase activity in H9C2 cells after siAGGF1 with or without siZEB1 (n=3/group, **P<0.01).

(b) Real-time RT-PCR analysis for *miR-183-5p* expression in H9C2 cells after siAGGF1 with or without siZEB1. (n=3/group, **P<0.01).

Data are shown as mean ± s.d. from at least three independent experiments. Statistical analysis was carried out by a Student’s two-tailed t-test.

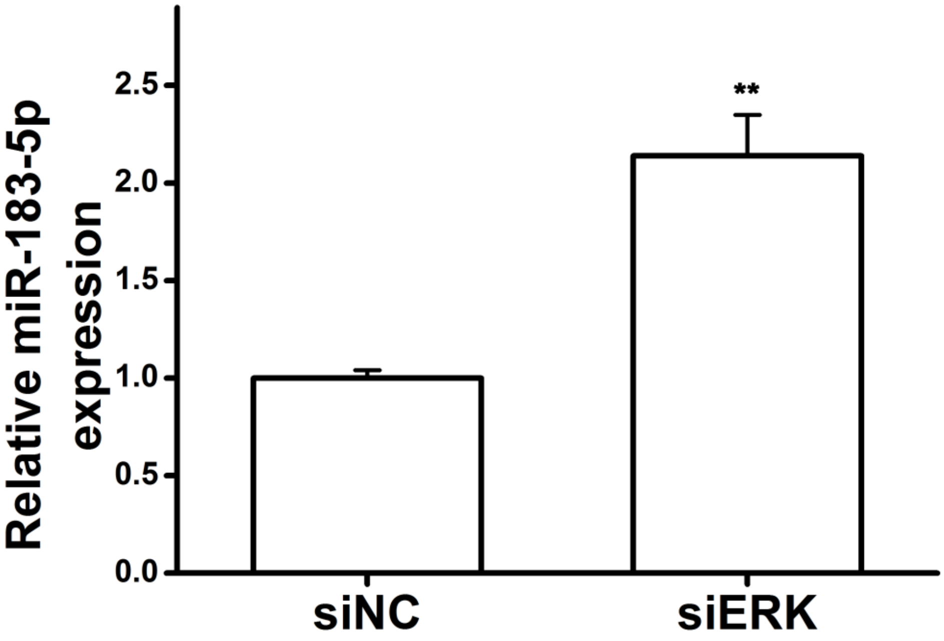

Supplementary Fig 13 Real-time RT-PCR analysis for *miR-183-5p* expression levels in H9C2 cells transfected with ERK specific siRNA (siERK) or control siRNA (siNC).

Data are shown as mean ± s.d. from at least three independent experiments (n=3/group, ***P*<0.01). Statistical analysis was carried out by a Student's two-tailed t-test.

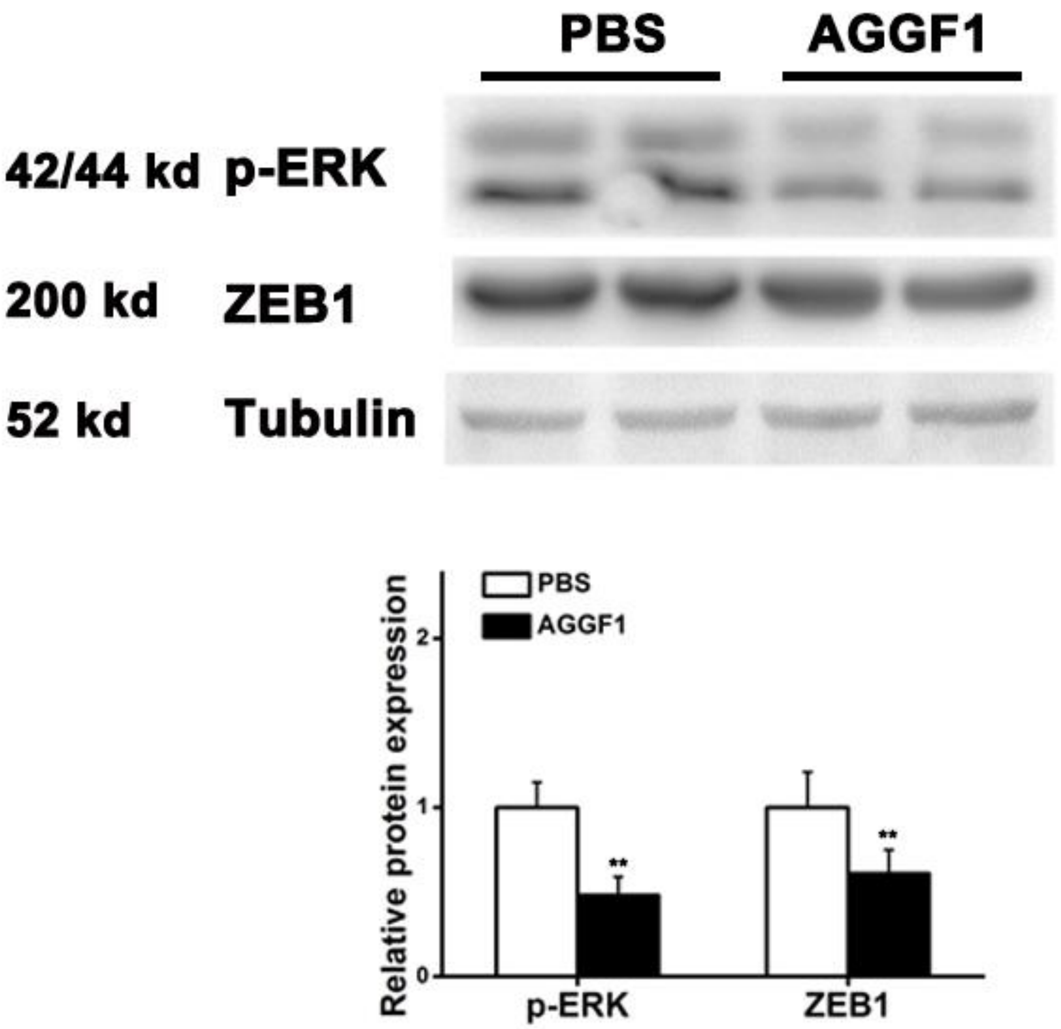

Supplementary Fig 14 Western blot analysis for the activation of ERK1/2 and ZEB1 expression in H9C2 cells treated with ISO with or without AGGF1 for 48 h. Data are shown as mean ± s.d. from at least three independent experiments (n=6/group, ***P*<0.01). Statistical analysis was carried out by a Student's two-tailed t-test.

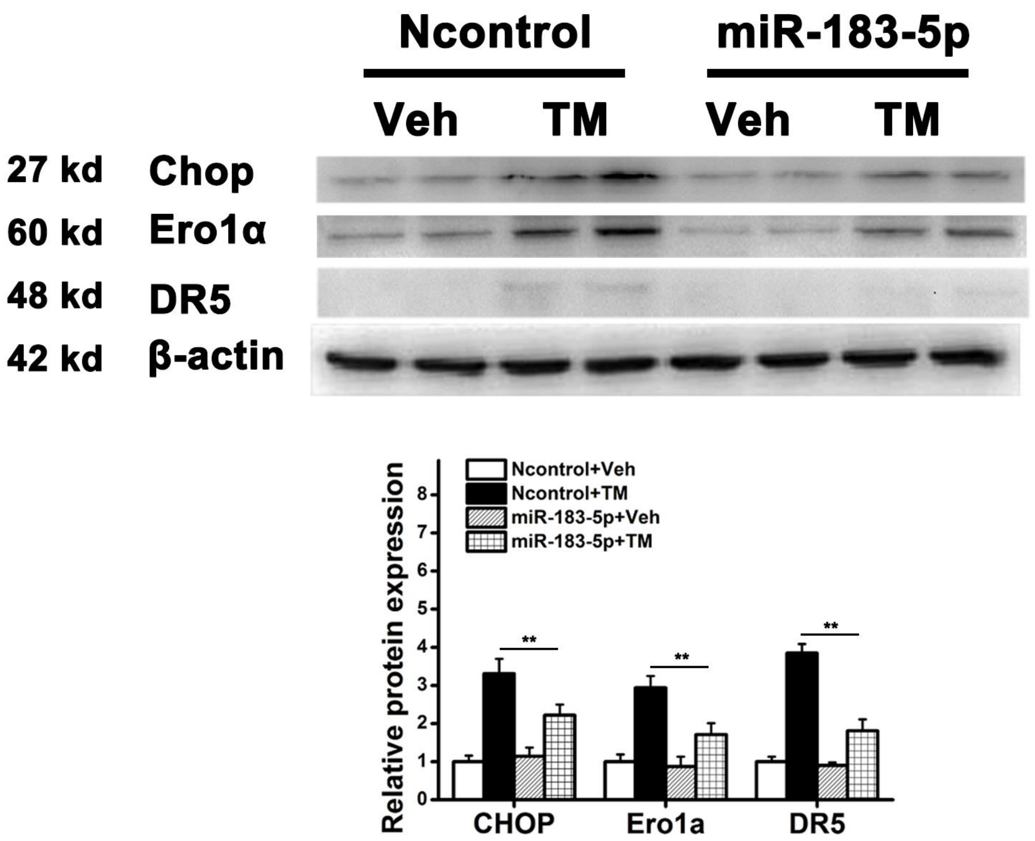

Supplementary Figure 15 ***MiR-183-5p*** **mimics reduces ER stress activator Tunicamycin (TM)-induced expression of CHOP, Ero1α, and DR5.** Data are shown as mean ± s.d. from at least three independent experiments (n=6/group, $^{**}P < 0.01$). Statistical analysis was carried out by a Student's two-tailed t-test.

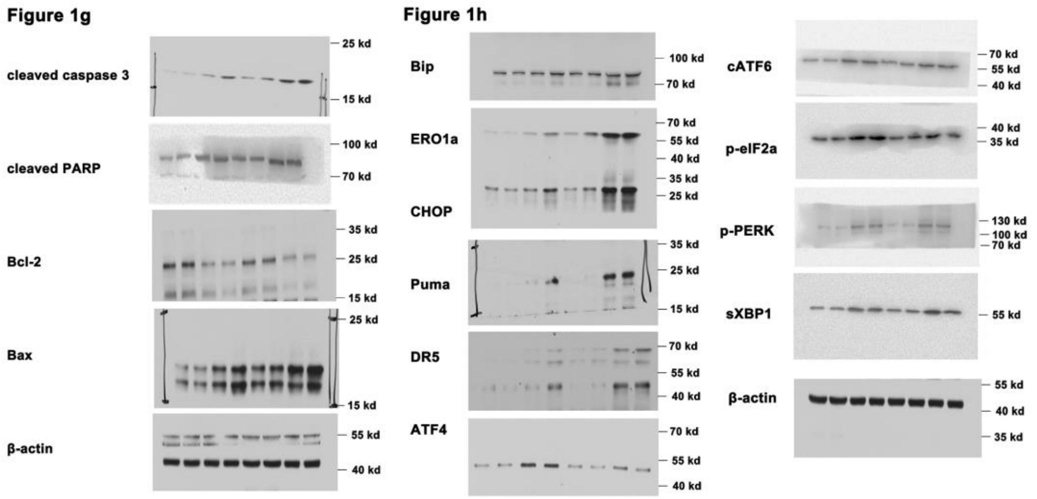

Supplementary Figure 16 Original Western blot images for Figure 1g and 1h.

Figure 2b

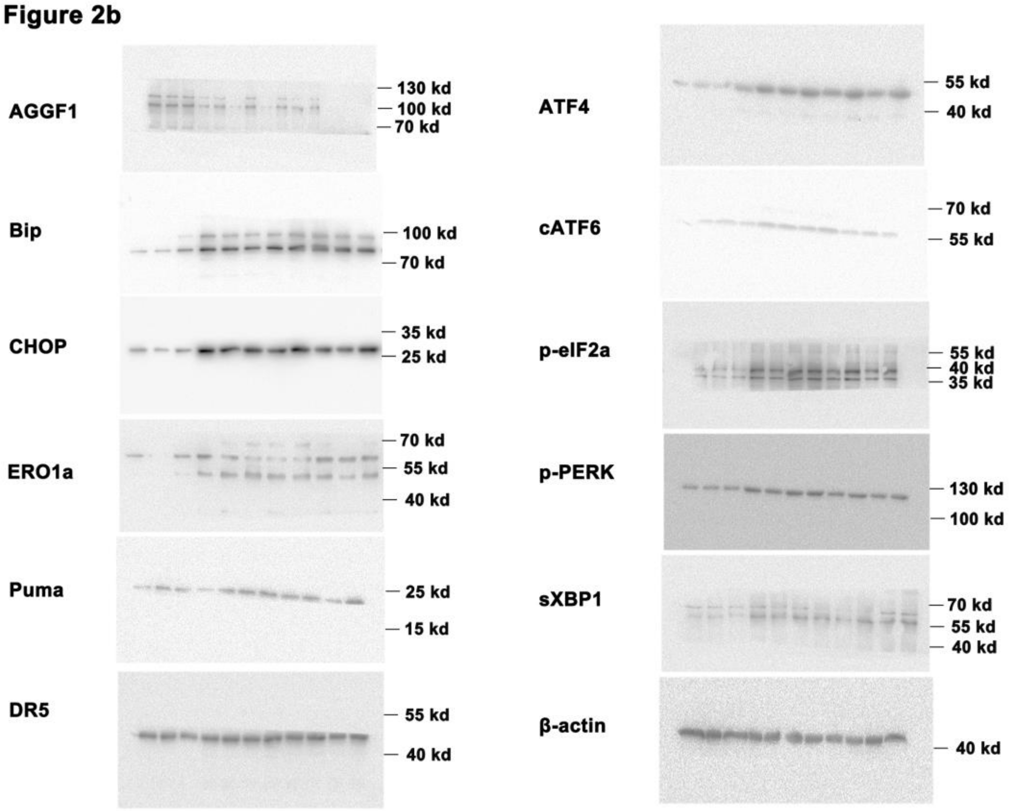

Figure 2e

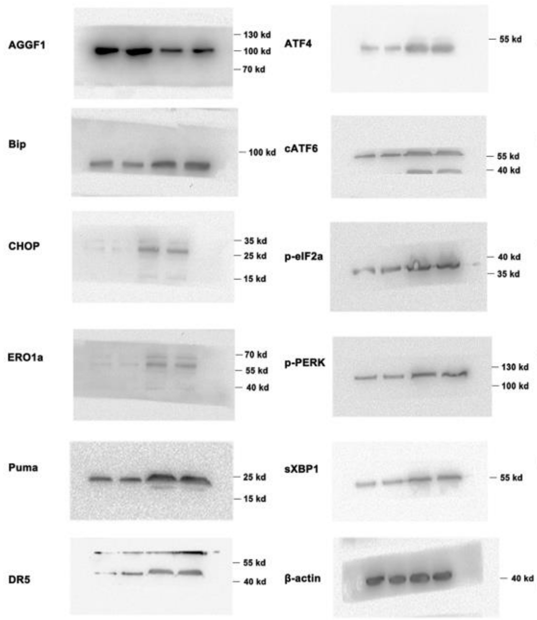

Figure 2g

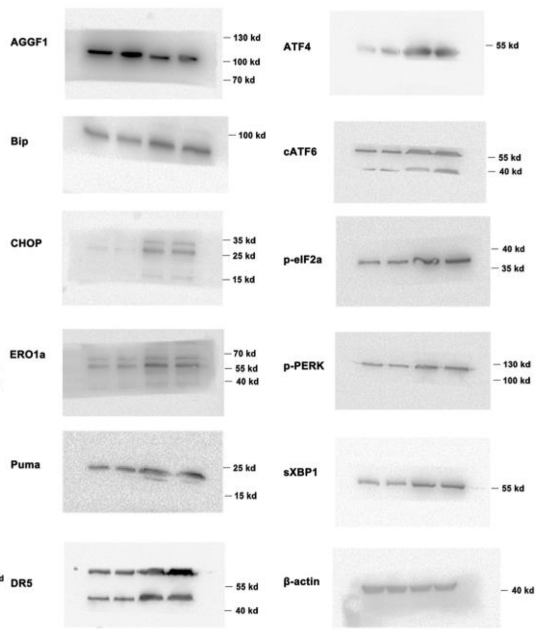

Supplementary Figure 17 Original Western blot images for Figures 4a, 4d, 4f and 5e.

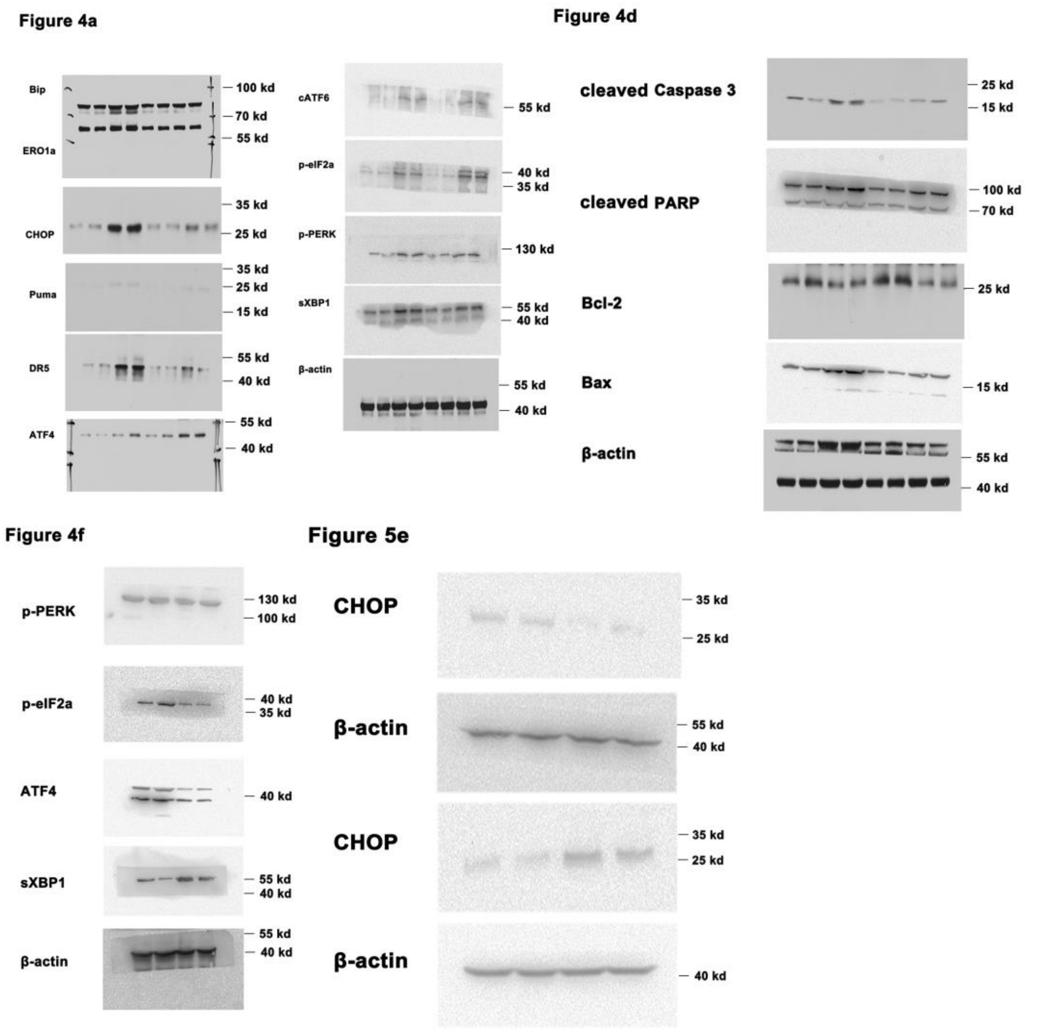

Supplementary Figure 18 Original Western blot images for Figures 4a, 4d, 4f and 5e.

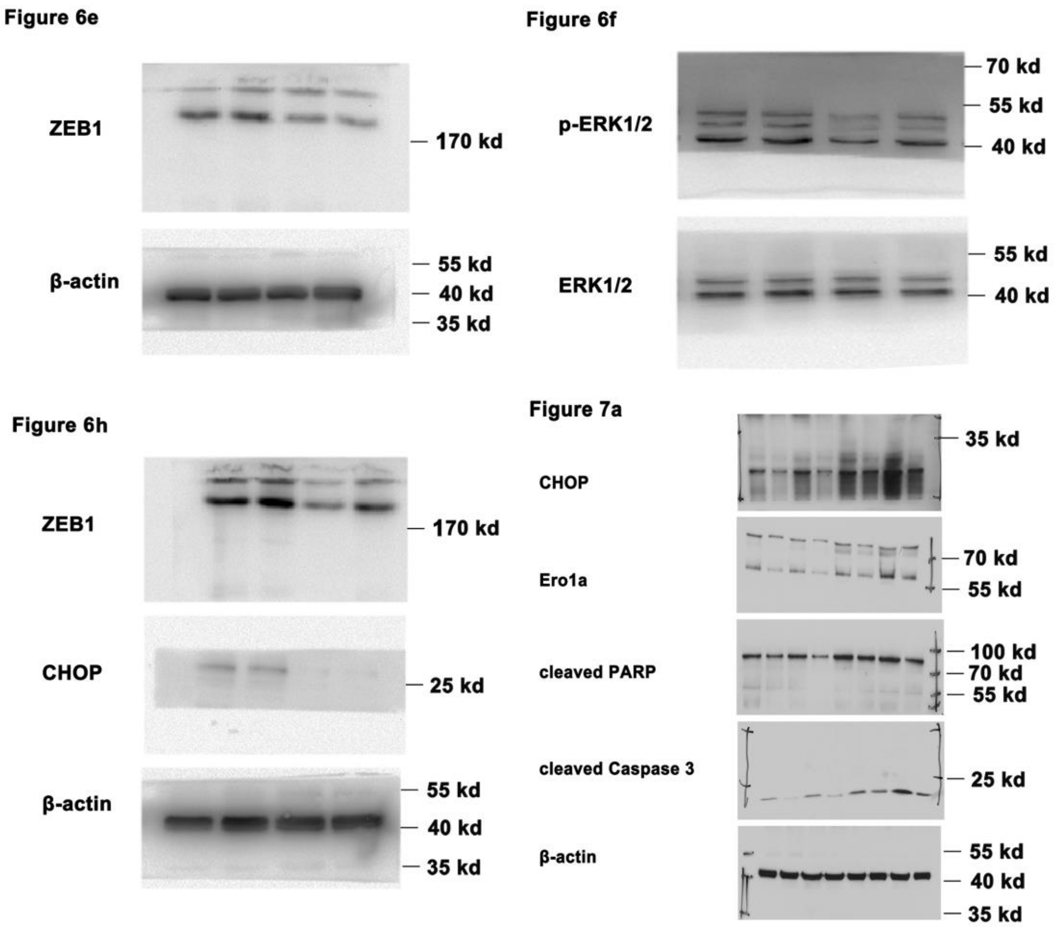

Supplementary Figure 19 Original Western blot images for Figures 6e, 6f, 6h and 7a.

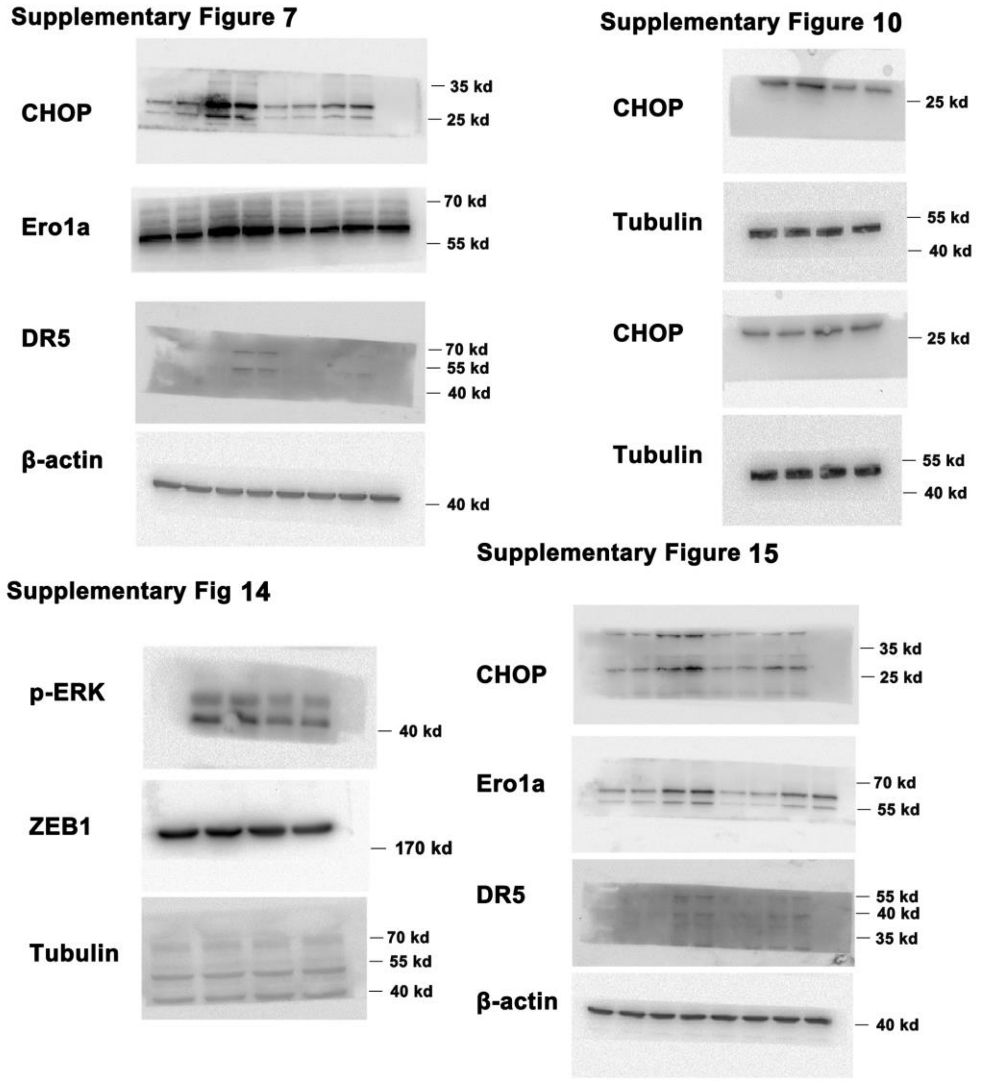

Supplementary Figure 20 Original Western blot images for Figures 7, 10, 14 and 15.

Supplementary Table 1 Sequences of primers used for Real-time RT-PCR analysis.

Gene	Sequence
Aggf1	F: CAAGCTCCTTGGGCGATTTCA
	R: GCATAACTCCCGCTGTCTATGT
ACTB	F: TTGCACATGCCGGAGCCGTT
	R: CACATGCCGGAGCCGTTGTA
ATF4	F: CCTGAACAGCGAAGTGTTGG
	R: TGGAGAACCCATGAGGTTTCAA
CHOP	F: CTCGCTCTCCAGATTCCAGTC
	R: CTTCATGCGTTGCTTCCCA
ERo1α	F: GTTAGTGGTTACCTGGACGACT
	R: CAGAATACTTGTAGCTCGCAGAC
Xbp1	F: CCTTGTGGTTGAGAACCAGG
	R: GTGTCAGAGTCCATGGGA
